# Genetically modified E. Coli secreting melanin (E.melanin) activates the astrocytic PSAP-GPR37L1 pathway and mitigates the pathogenesis of Parkinson’s disease

**DOI:** 10.1186/s12951-024-02955-x

**Published:** 2024-11-10

**Authors:** Weixian Kong, Yu Liu, Pu Ai, Yong Bi, Chaoguang Wei, Xiaoyang Guo, Zhenyu Cai, Ge Gao, Peng Hu, Jialin Zheng, Jianhui Liu, Minfeng Huo, Yuting Guan, Qihui Wu

**Affiliations:** 1 Shanghai Research Institute for Intelligent Autonomous Systems, Shanghai Key Laboratory of Anesthesiology and Brain Functional Modulation, Clinical Research Center for Anesthesiology and Perioperative Medicine, Translational Research Institute of Brain and Brain-Like Intelligence, Shanghai Fourth People’s Hospital Affiliated to Tongji University School of Medicine, State Key Laboratory of Cardiology and Medical Innovation Center, Shanghai East Hospital, School of Medicine, Tongji University, Shanghai, 200092 China; 2 Department of Neurology, Shanghai University of Medicine & Health Sciences Affiliated Zhoupu Hospital, 1500 Zhouyuan Road, Pudong New Area, Shanghai, 201318 China; 3 Key Laboratory of Exploration and Utilization of Aquatic Genetic Resources, Ministry of Education, Shanghai Ocean University, Shanghai, 201306 China; 4 Center for Translational Neurodegeneration and Regenerative Therapy, Tongji Hospital, Shanghai Frontiers Science Center of Nanocatalytic Medicine, The Institute for Biomedical Engineering & Nano Science, School of Medicine, Tongji University School of Medicine, Tongji University, Shanghai, 200092 China; 5 Cancer Center, Tenth Peoples Hospital of Tongji University, Shanghai, 200070 China; 6 Department of Anaesthesiology, School of Medicine, Tongji Hospital, Tongji University, Shanghai, China; 7 Shanghai Frontiers Science Center of Nanocatalytic Medicine, School of Medicine, Shanghai Tenth People’s Hospital, Tongji University, Shanghai, 200072 China; 8 Shanghai Frontiers Science Center of Genome Editing and Cell Therapy, Shanghai Key Laboratory of Regulatory Biology, Institute of Biomedical Sciences, School of Life Sciences, East China Normal University, Shanghai, 200241 China; 9 College of Pharmacy, Ningxia Medical University, Ningxia Hui Autonomous Region, Yinchuan, 750004 China

**Keywords:** Melanin, Parkinson’s disease, Dopamine neuron, Astrocyte, PSAP-GPR37L1 pathway

## Abstract

The characteristic neuropathology of Parkinson’s disease (PD) involves the abnormal accumulation of phosphorylated α-synuclein (αSyn), as well as a significant decrease in neuromelanin (NM) levels within dopamine neurons (DaNs). Unlike αSyn aggregates, the relationship between NM levels and PD pathogenesis is not well understood. In this study, we engineered an E. coli MG1655 strain to produce exosomes containing melanin (E.melanin), and investigated its potential neuroprotective effects on DaNs in the context of PD. By employing a combination of cell cultures, biochemical studies, single nuclear RNA sequencing (snRNA seq), and various in vivo validations, we found that administration of E.melanin effectively alleviated DaNs loss and improved motor behavior impairments observed in both pharmacological and transgenic PD mouse models. Mechanistically, snRNA seq data suggested that E.melanin activated the PSAP-GPR37L1 signaling pathway specifically within astrocytes, leading to a reduction in astrocytic engulfment of synapses. Notably, activation of the GPR37L1 receptor using Tx14(A) peptide successfully rescued motor defects as well as protected against DaNs degeneration in mice with PD. Overall, our findings provide novel insights into understanding the molecular mechanisms underlying melanin’s protective effects on DaNs in PD while offering potential strategies for manipulating and treating its pathophysiological progression.

**Supplementary Information:**

The online version contains supplementary material available at 10.1186/s12951-024-02955-x.

## Introduction

Parkinson’s disease (PD) is a prevalent and intricate neurodegenerative disorder that is progressive and associated with aging [1]. The clinical management of PD involves symptomatic treatment with medications that either augment dopamine levels or directly activate dopamine receptors [2]. However, this approach only provides relief for motor dysfunction without halting or reversing the progressive degeneration of dopaminergic neurons (DaNs) in the substantia nigra pars compacta (SNpc) [3].

Essentially, the neuropathological hallmark of PD involves the presence of abnormal inclusions called Lewy bodies (LBs) and Lewy neurites (LNs) within neural cells [4], which mainly consist of aberrant aggregates of phosphorylated α-synuclein (αSyn) [5]. Another prominent histological characteristic of PD is the reduction in neuromelanin (NM), a black-brown pigment present in dopamine-producing neurons [6, 7]. In healthy individuals, NM levels gradually increase with normal aging, but patients with PD exhibit a significant decrease in NM levels [8, 9]. NM exerts both protective and toxic effects within the body [10, 11]. On one hand, it acts protectively by reducing oxidative damage within cells through the removal of excessive dopamine and chelation of toxic metal ions such as iron, zinc, copper, manganese, among others. On the other hand, its insolubility can lead to progressive accumulation within cells impairing cellular transport and signal transmission. This accumulation can result in mitochondrial respiratory damage, reduction in cellular metabolic activity, ultimately leading to neuronal dysfunction and degeneration [12, 13]. Unlike αSyn aggregates, the pathophysiological association between NM levels and PD is not well understood.

The limited understanding of NM primarily due to the challenges associated with elucidating its chemical structures, low solubility, and limited availability for research purposes, which was mainly obtained from postmortem human brains [14]. Unlike humans, laboratory animal species commonly used in experimental research, such as rodents, do not possess NM [15]. Consequently, the significance of NM has been unexpectedly overlooked in animal models of PD in vivo [16]. These factors contribute to difficulties in comprehensively understanding NM. Nonetheless, Vila and colleagues demonstrated that overexpression of human tyrosine hydroxylase gene led to age-dependent production of human-like NM specifically within DaNs in rats [17], suggesting that exogenous melanin precursor substrate/enzymes can potentially increase NM levels in the brain. However, intracellular neuromelanin levels may set the threshold for the initiation of PD, since direct overexpression of melanin leads to neuronal death in these animals [17]. Therefore, the utilization of melanin in neurodegeneration associated with PD remains a formidable challenge that necessitates persistent efforts.

In the present study, we systematically investigated the neuroprotective roles of melanin in the degeneration of DaNs. We hypothesized that E.coli-derived melanin-containing exosomes not only enhanced its solubility but also increased the resilience of DaNs, ultimately attenuating αSyn-induced pathogenesis in mice with PD.

## Results

### Construction and characterization of a genetically engineered E. Coli capable of secreting melanin

**Fig. 1:**
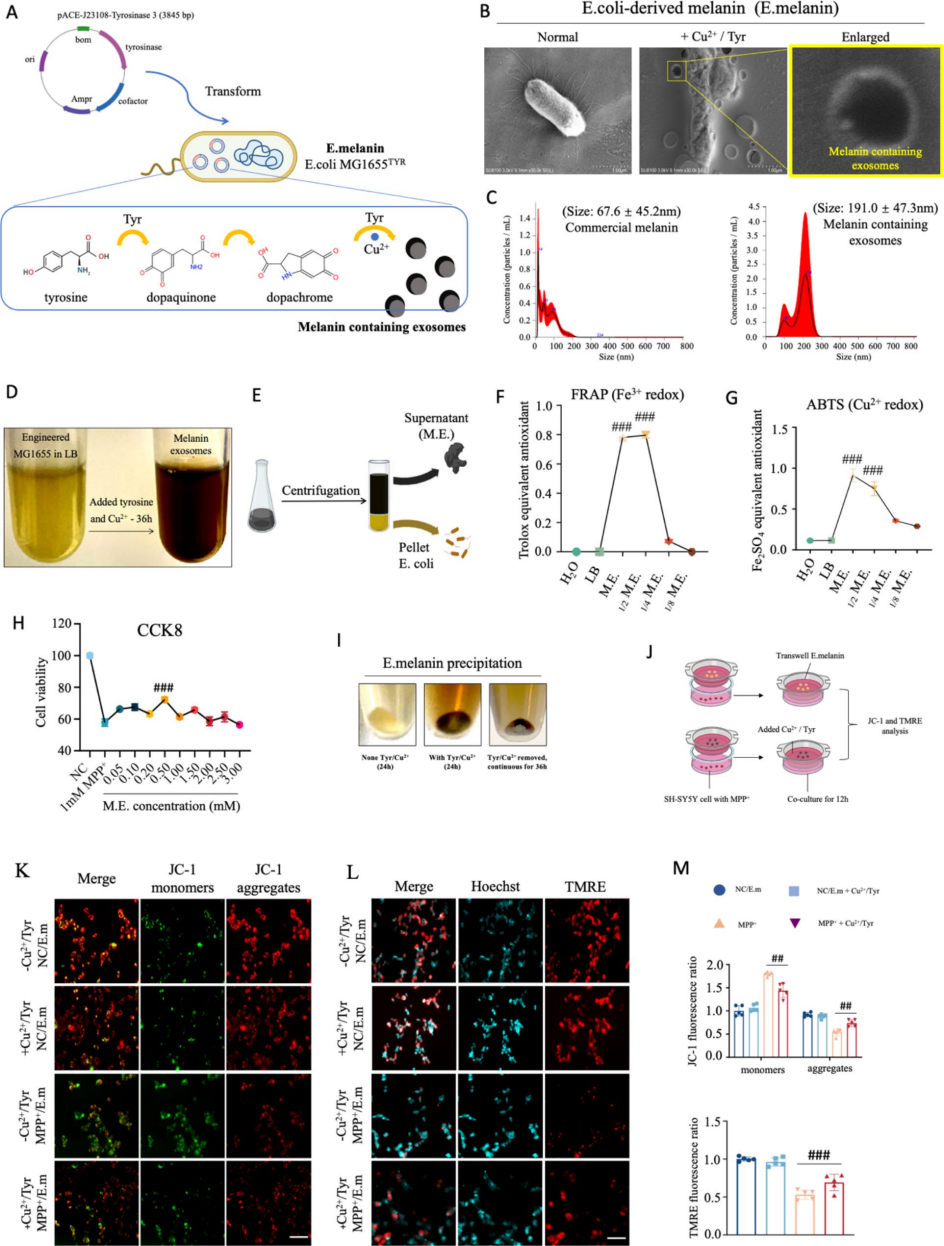
Construction and characterization of a genetically engineered E. coli capable of secreting melanin. (**A**) Cartoons illustrate the genetic manipulation of E. coli that capable of secreting melanin in exosomes. (**B**) Typical scanning electron microscopy (SEM) graphs of E. coli with or without Cu^2+^/tyrosine incubation. In the presence of Cu^2+^/tyrosine, the dark colored melanin was released into extracellular via exosome (enlarged image). (**C**) NTA analysis of commercial (left panel) and E. coli-derived (right panel) melanin. (**D**) and (**E**) Induction and purification of E.melanin via ultracentrifuge. After centrifugation, the E.melanin was remained in the supernatant. (**F**) FRAP analysis of antioxidative activity. One-way ANOVA test, ***p < 0.001. (**G**) ABTS analysis of antioxidative activity. One-way ANOVA test, ***p < 0.001. (**H**) CCK8 assay of SH-SY5Y cells treated with MPP + and purified melanin. One-way ANOVA test, ***p < 0.001. (**I**) Continuous release of E.melanin after Cu^2+^/tyrosine incubated for 24 h. When E. coli was incubated with Cu^2+^/tyrosine for 24 h, they can continuously synthesis and release E.melanin after Cu^2+^/tyrosine removal. (**J**) Schematic cartoons illustrate the co-culture of E. coli and SH-SY5Y cells in the trans-well chambers. The E. coli was pre-incubated with Cu^2+^/tyrosine for 24 h, then purified via centrifuge and cultured in the above chamber, where SH-SY5Y cells were cultured in the bottom. (**K**) Typical JC-1 staining of MPP^+^-treated SH-SY5Y cells that were co-cultured with E. coli with distinct pre-treatments. Scale bar: 50 μm. (**L**) Typical TMRE staining of MPP^+^-treated SH-SY5Y cells that were co-cultured with E. coli with distinct pre-treatments. Scale bar: 50 μm. (**M**) Quantifications of JC-1 monomers and aggregates and TMRE fluorescence intensity. One-way ANOVA test, ns, no significant; **p < 0.01; ***p < 0.001

Recently, we have genetically engineered a E. coli MG1655 strain that was capable of secreting melanin, referred to as E.melanin (Fig. 1A) [18]. Subsequently, a comprehensive characterization of its property was conducted. Initially, scanning electron microscopy (SEM) was employed to visualize the morphology of E. coli in the presence or absence of Cu^2+^ and tyrosine (Tyr), which served as triggers for biogenesis and secretion of melanin-containing exosomes (Fig. 1B). Surprisingly, when Cu^2+^/Tyr were present, dark-colored extracellular vesicles or exosomes were observed surrounding E. coli, indicating successful induction of melanin that was encapsulated within these exosomes (Fig. 1B, right two panels). In contrast to commercialized melanin with low water solubility (Supplementary Fig. 1A and 1B), the E. coli-derived exosomal melanin exhibited uniform particle size as confirmed by nanoparticle tracking analysis (NTA) assay (Fig. 1C).

Melanin derived from various sources exhibit significant antioxidant activity, including a potent ability to scavenge or quench free radicals and reactive oxygen species (ROS) [19]. Subsequently, we performed ultracentrifugation to isolate and purify E. coli-derived exosomal melanin (referred to as M.E.) (Fig. 1D and E). The purified M.E. demonstrated a dose-dependent antioxidant property in the FRAP and ABTS assays respectively (Fig. 1F and G). Based on the CCK8 assay, 1 mM concentration of 1-methyl-4-phenyl-pyridinium (MPP^+^) was used for further investigations (Supplementary Fig. 1C). In addition, the treatment of M.E. had no significant cytotoxic effect on cell viability up to 2.5 mM (Supplementary Fig. 1D) since higher concentration of melanin is toxic [20]. Consistently, M.E. exhibited a significant dose-dependent increase in neuroprotection when exposed to MPP^+^ in SH-SY5Y cell (Fig. 1H), which serves as an extensively used in vitro model for studying dopaminergic neuron-like behaviors in response to neurotoxins associated with PD development [21]. However, commercialized melanin only increased cell viability from 60.10 to 61.50% (Supplementary Fig. 1E), indicating that the biological activity of melanin relies on its structural stability. Additionally, after treating E.melanin with Cu^2+^/Tyr for 24 h followed by medium replacement without further induction, continuous secretion of M.E. was observed (Fig. 1I). Subsequently, this pre-treated E.melanin was co-cultured with SH-SY5Y cells (Fig. 1J). Consistently, SH-SY5Y cells that co-cultured with Cu^2+^/Tyr pre-treated E.melanin displayed substantial protective effects against MPP^+^ treatment as evidenced by JC-1 and TMRE staining assays respectively (Fig. 1K-M).

Then, E.melanin was encapsulated in an inulin gel and administered into mice via intragastric infusion every other day for two weeks (Supplementary Fig. 2A and 2B). Mice treated with E.melanin showed a slight decrease in body weight which gradually recovered to that of controls (Supplementary Fig. 2C), indicating mild discomfort experienced by the mice. Furthermore, qPCR results also revealed a slight increase in inflammatory genes in intestines treated with E.melanin (Supplementary Fig. 2D). Additionally, immunocytochemistry staining was conducted, and more melanin positive cells were observed in E.melanin administered gut, striatum, and midbrain (Supplementary Fig. 2E). However, in the Hematoxylin and Eosin (H&E) staining, no significant damage was observed in heart, liver, spleen, lung, kidney, gut, and brain tissues from mice with or without E.melanin infusion (Supplementary Fig. 2F and 2G, and Table 1). Importantly, most of the Cy5-labeled E.melanin were localized within brain after tail vein infusion (Supplementary Fig. 3). Consistently, western blot analysis of apoptotic proteins such as Caspase-3, cleaved Caspase-3, Bcl-2 and Bax also demonstrated no significant changes in the tissues analyzed (Supplementary Fig. 4).

**Table 1:** Blood cell routine analysis

Number: Gel 1 Type: Whole blood				
Parameter	Abbr.	Result	Unit	Reference
White blood cell count	WBC	7.4	10^9/L	0.8–10.6
Lymphocyte count	Lymph#	6	10^9/L	0.6–8.9
Monocytes count	Mon#	0.1	10^9/L	0.04–1.4
Neutrophils count	Gran#	1.3	10^9/L	0.23–3.6
Lymphocyte percentage	Lymph%	80.5	%	40–92
Monocytes percentage	Mon%	2.5	%	0.9–18
Neutrophils percentage	Gran%	17	%	6.5–50
Red blood cell count	RBC	10.56	10^12/L	6.5–11.5
Hemoglobin	HGB	161	g/L	110–165
Hematocrit	HCT	49.1	%	35–55
Mean corpuscular volume	MCV	46.5	fL	41–55
Mean corpuscular hemoglobin	MCH	15.2	pg	13–18
Platelet count	PLT	1950	10^9/L	400–1600
Mean platelet volume	MPV	6.3	fL	4.0-6.2

Number: Gel 2 Type: Whole blood				
Parameter	Abbr.	Result	Unit	Reference
White blood cell count	WBC	3.2	10^9/L	0.8–10.6
Lymphocyte count	Lymph#	2.5	10^9/L	0.6–8.9
Monocytes count	Mon#	0.1	10^9/L	0.04–1.4
Neutrophils count	Gran#	0.6	10^9/L	0.23–3.6
Lymphocyte percentage	Lymph%	78.4	%	40–92
Monocytes percentage	Mon%	2.6	%	0.9–18
Neutrophils percentage	Gran%	19	%	6.5–50
Red blood cell count	RBC	10.7	10^12/L	6.5–11.5
Hemoglobin	HGB	162	g/L	110–165
Hematocrit	HCT	51.3	%	35–55
Mean corpuscular volume	MCV	48	fL	41–55
Mean corpuscular hemoglobin	MCH	15.1	pg	13–18
Platelet count	PLT	693	10^9/L	400–1600
Mean platelet volume	MPV	6.2	fL	4.0-6.2

Number: Gel 3 Type: Whole blood				
Parameter	Abbr.	Result	Unit	Reference
White blood cell count	WBC	5.9	10^9/L	0.8–10.6
Lymphocyte count	Lymph#	4.8	10^9/L	0.6–8.9
Monocytes count	Mon#	0.1	10^9/L	0.04–1.4
Neutrophils count	Gran#	1	10^9/L	0.23–3.6
Lymphocyte percentage	Lymph%	80.8	%	40–92
Monocytes percentage	Mon%	2.1	%	0.9–18
Neutrophils percentage	Gran%	17.1	%	6.5–50
Red blood cell count	RBC	10.09	10^12/L	6.5–11.5
Hemoglobin	HGB	152	g/L	110–165
Hematocrit	HCT	47.7	%	35–55
Mean corpuscular volume	MCV	47.3	fL	41–55
Mean corpuscular hemoglobin	MCH	15	pg	13–18
Platelet count	PLT	1391	10^9/L	400–1600
Mean platelet volume	MPV	5.6	fL	4.0-6.2

Number: Gel 4 Type: Whole blood				
Parameter	Abbr.	Result	Unit	Reference
White blood cell count	WBC	5.3	10^9/L	0.8–10.6
Lymphocyte count	Lymph#	4.4	10^9/L	0.6–8.9
Monocytes count	Mon#	0.1	10^9/L	0.04–1.4
Neutrophils count	Gran#	0.8	10^9/L	0.23–3.6
Lymphocyte percentage	Lymph%	82.7	%	40–92
Monocytes percentage	Mon%	2	%	0.9–18
Neutrophils percentage	Gran%	15.3	%	6.5–50
Red blood cell count	RBC	10.39	10^12/L	6.5–11.5
Hemoglobin	HGB	156	g/L	110–165
Hematocrit	HCT	49.3	%	35–55
Mean corpuscular volume	MCV	47.5	fL	41–55
Mean corpuscular hemoglobin	MCH	15	pg	13–18
Platelet count	PLT	1179	10^9/L	400–1600
Mean platelet volume	MPV	7.2	fL	4.0-6.2

Number: Gel 5 Type: Whole blood				
Parameter	Abbr.	Result	Unit	Reference
White blood cell count	WBC	7	10^9/L	0.8–10.6
Lymphocyte count	Lymph#	5.7	10^9/L	0.6–8.9
Monocytes count	Mon#	0.2	10^9/L	0.04–1.4
Neutrophils count	Gran#	1.1	10^9/L	0.23–3.6
Lymphocyte percentage	Lymph%	82	%	40–92
Monocytes percentage	Mon%	2.6	%	0.9–18
Neutrophils percentage	Gran%	15.4	%	6.5–50
Red blood cell count	RBC	11.04	10^12/L	6.5–11.5
Hemoglobin	HGB	165	g/L	110–165
Hematocrit	HCT	50.3	%	35–55
Mean corpuscular volume	MCV	45.6	fL	41–55
Mean corpuscular hemoglobin	MCH	14.9	pg	13–18
Platelet count	PLT	1403	10^9/L	400–1600
Mean platelet volume	MPV	6.8	fL	4.0-6.2

Number: E.melanin 1 Type: Whole blood				
Parameter	Abbr.	Result	Unit	Reference
White blood cell count	WBC	4.6	10^9/L	0.8–10.6
Lymphocyte count	Lymph#	3.3	10^9/L	0.6–8.9
Monocytes count	Mon#	0.2	10^9/L	0.04–1.4
Neutrophils count	Gran#	1.1	10^9/L	0.23–3.6
Lymphocyte percentage	Lymph%	71.6	%	40–92
Monocytes percentage	Mon%	3.9	%	0.9–18
Neutrophils percentage	Gran%	24.5	%	6.5–50
Red blood cell count	RBC	10.21	10^12/L	6.5–11.5
Hemoglobin	HGB	163	g/L	110–165
Hematocrit	HCT	46.5	%	35–55
Mean corpuscular volume	MCV	45.6	fL	41–55
Mean corpuscular hemoglobin	MCH	15.9	pg	13–18
Platelet count	PLT	1702	10^9/L	400–1600
Mean platelet volume	MPV	5.8	fL	4.0-6.2

Number: E.melanin 2 Type: Whole blood				
Parameter	Abbr.	Result	Unit	Reference
White blood cell count	WBC	5.6	10^9/L	0.8–10.6
Lymphocyte count	Lymph#	4.2	10^9/L	0.6–8.9
Monocytes count	Mon#	0.1	10^9/L	0.04–1.4
Neutrophils count	Gran#	1.3	10^9/L	0.23–3.6
Lymphocyte percentage	Lymph%	73.7	%	40–92
Monocytes percentage	Mon%	2.5	%	0.9–18
Neutrophils percentage	Gran%	23.8	%	6.5–50
Red blood cell count	RBC	10.66	10^12/L	6.5–11.5
Hemoglobin	HGB	159	g/L	110–165
Hematocrit	HCT	49.1	%	35–55
Mean corpuscular volume	MCV	46.1	fL	41–55
Mean corpuscular hemoglobin	MCH	14.9	pg	13–18
Platelet count	PLT	2002	10^9/L	400–1600
Mean platelet volume	MPV	6.4	fL	4.0-6.2

Number: E.melanin 3 Type: Whole blood				
Parameter	Abbr.	Result	Unit	Reference
White blood cell count	WBC	3.3	10^9/L	0.8–10.6
Lymphocyte count	Lymph#	2.7	10^9/L	0.6–8.9
Monocytes count	Mon#	0.1	10^9/L	0.04–1.4
Neutrophils count	Gran#	0.5	10^9/L	0.23–3.6
Lymphocyte percentage	Lymph%	81.3	%	40–92
Monocytes percentage	Mon%	2.2	%	0.9–18
Neutrophils percentage	Gran%	16.5	%	6.5–50
Red blood cell count	RBC	10.98	10^12/L	6.5–11.5
Hemoglobin	HGB	163	g/L	110–165
Hematocrit	HCT	51.2	%	35–55
Mean corpuscular volume	MCV	46.7	fL	41–55
Mean corpuscular hemoglobin	MCH	14.8	pg	13–18
Platelet count	PLT	1173	10^9/L	400–1600
Mean platelet volume	MPV	6.9	fL	4.0-6.2

Number: E.melanin 4 Type: Whole blood				
Parameter	Abbr.	Result	Unit	Reference
White blood cell count	WBC	6.4	10^9/L	0.8–10.6
Lymphocyte count	Lymph#	5.3	10^9/L	0.6–8.9
Monocytes count	Mon#	0.1	10^9/L	0.04–1.4
Neutrophils count	Gran#	1	10^9/L	0.23–3.6
Lymphocyte percentage	Lymph%	82.7	%	40–92
Monocytes percentage	Mon%	2	%	0.9–18
Neutrophils percentage	Gran%	15.3	%	6.5–50
Red blood cell count	RBC	9.98	10^12/L	6.5–11.5
Hemoglobin	HGB	151	g/L	110–165
Hematocrit	HCT	46.2	%	35–55
Mean corpuscular volume	MCV	46.3	fL	41–55
Mean corpuscular hemoglobin	MCH	15.1	pg	13–18
Platelet count	PLT	1144	10^9/L	400–1600
Mean platelet volume	MPV	6	fL	4.0-6.2

Number: E.melanin 5 Type: Whole blood				
Parameter	Abbr.	Result	Unit	Reference
White blood cell count	WBC	6.2	10^9/L	0.8–10.6
Lymphocyte count	Lymph#	4.7	10^9/L	0.6–8.9
Monocytes count	Mon#	0.2	10^9/L	0.04–1.4
Neutrophils count	Gran#	1.3	10^9/L	0.23–3.6
Lymphocyte percentage	Lymph%	76.6	%	40–92
Monocytes percentage	Mon%	2.5	%	0.9–18
Neutrophils percentage	Gran%	20.9	%	6.5–50
Red blood cell count	RBC	10.99	10^12/L	6.5–11.5
Hemoglobin	HGB	166	g/L	110–165
Hematocrit	HCT	50	%	35–55
Mean corpuscular volume	MCV	45.5	fL	41–55
Mean corpuscular hemoglobin	MCH	15.1	pg	13–18
Platelet count	PLT	1608	10^9/L	400–1600
Mean platelet volume	MPV	6.3	fL	4.0-6.2

Collectively, these findings indicate that genetically engineered E.melanin exhibits robust antioxidant activity and possesses potential for ameliorating dopaminergic neurodegeneration associated with PD.

### Administration of E.melanin alleviated motor defects and dopamine neuron loss in pharmacology-induced PD mice

**Fig. 2:**
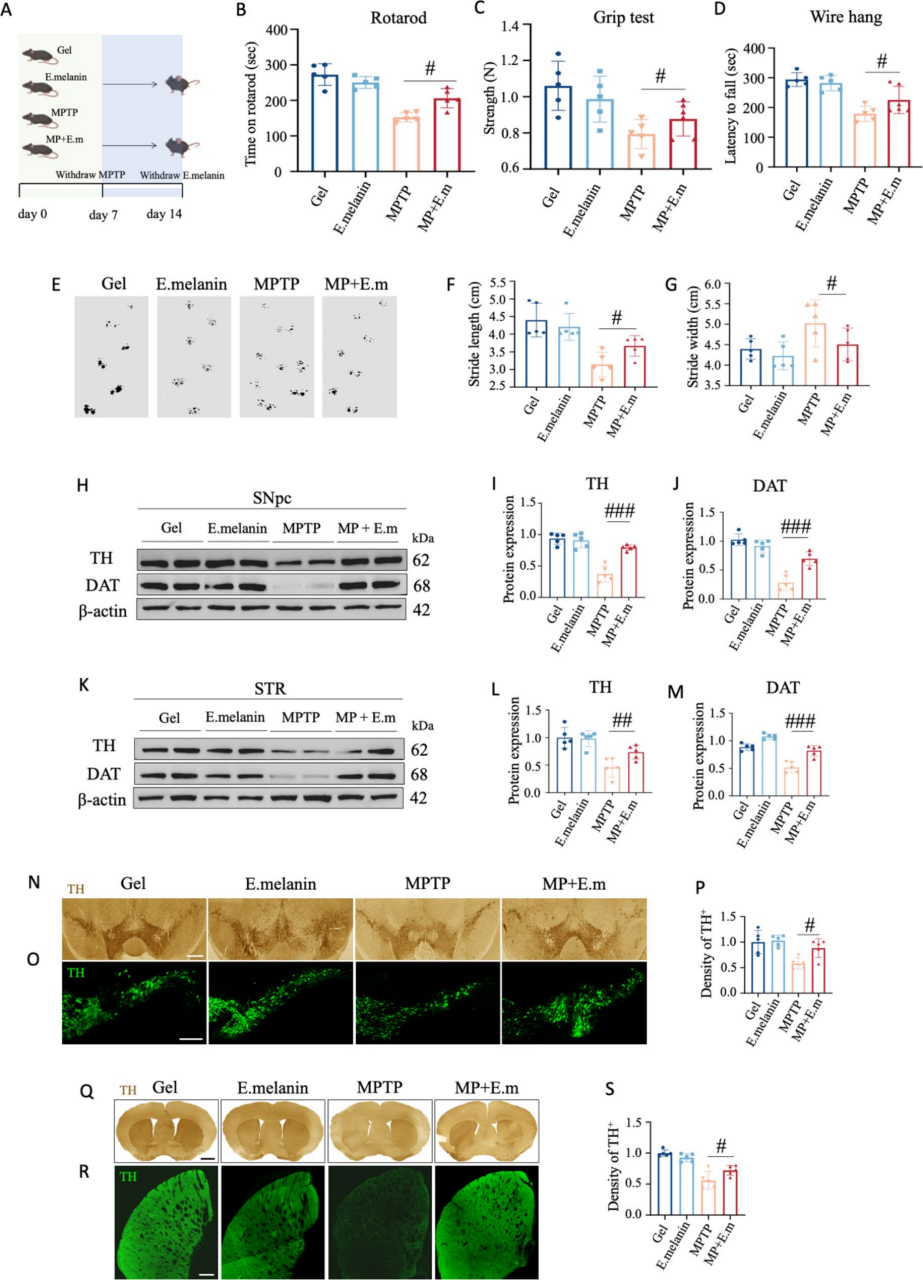
Administration of E.melanin partially recovered motor defects and DaNs loss in pharmacology-induced PD mice. (**A**) Cartoons illustrate the time points of MPTP and E.melanin administration. MPTP was intraperitoneally (i.p.) injected at 25 mg/kg for seven days to induce PD mouse model, where inulin gel encapsulated E.melanin was orally administered at 1*10^8^ CFU for seven times with two-day intervals between each administration. (**B**)-(**D**) Motor behavior tests in MPTP-injected mice with or without E.melanin treatment. E.melanin partially rescued MPTP-induced motor defects in rotarod test (**B**), grip strength test (**C**), and wire hang test (**D**). (**E**) Typical graphs of gait analysis in MPTP-injected mice with or without E.melanin treatment. (**F**) and (**G**) Quantifications of stride length (**F**) and stride width (**G**). One-way ANOVA test, *p < 0.05. (**H**) Western blot analysis of TH and DAT in SNpc. (**I**) and (**J**) Quantifications of TH (**I**) and DAT (**J**) in SNpc. One-way ANOVA test, ns, no significant; ***p < 0.001. (**K**) Western blot analysis of TH and DAT in STR. (**L**) and (**M**) Quantifications of TH (**L**) and DAT (**M**) in STR. One-way ANOVA test, **p < 0.01; ***p < 0.001. (**N**) Typical immunohistochemistry staining of TH in SNpc. Scale bar: 500 μm. (**O**) Typical immunofluorescent staining of TH in SNpc. Scale bar: 100 μm. (**P**) Quantifications of TH in SNpc from panel (**O**). One-way ANOVA test, *p < 0.05; **p < 0.01. (**Q**) Typical immunohistochemistry staining of TH in STR. Scale bar: 500 μm. (**R**) Typical immunofluorescent staining of TH in STR. Scale bar: 100 μm. (**S**) Quantifications of TH in STR from panel (**R**). One-way ANOVA test, *p < 0.05

Next, we initially evaluated the neuroprotective potential of E.melanin in a mouse model of PD induced by neurotoxin. 10-week-old male C57BL/6 mice were used for all experiments. After one week of acclimatization, mice were received intraperitoneal injections (i.p.) of 1-methyl-4-phenyl-1,2,3,6-tetrahydropyridine (MPTP) at a dosage of 25 mg/kg for seven consecutive days to induce PD models [22]. Subsequently, mice were orally administered with 1*10^8^ CFU of E.melanin seven times with two-day intervals between each administration (Fig. 2A). From the behavioral analysis, motor coordination and balance deficits were observed in MPTP-injected mice through rotarod test, grip test, wire hang test as well as gait analysis; however, these motor impairments were partially alleviated by E.melanin administration (Fig. 2B and G).

Considering that dopaminergic neuronal loss in the nigrostriatal system is a major pathological characteristic of PD, we next examined whether E.melanin treatment protected against the loss of dopamine neurons (DaNs) in the SNpc and striatum (STR). As expected, E.melanin supplementation relieved the reduction of tyrosine hydroxylase (TH) and dopamine transporter (DAT) expression levels in SNpc and STR induced by MPTP via western blot assay (Fig. 2H-M). Consistently, there was a significant decrease in TH-positive dopaminergic signals observed through immunocytochemistry and immunofluorescent staining in SNpc and STR from PD mice; while this phenomenon was mitigated by E.melanin treatment compared to untreated PD mice (Fig. 2N-S). Moreover, MPTP-induced robust TH-positive neuron loss was also found within the intestine, and this reduction was partially rescued by E.melanin supplementation (Supplementary Fig. 5).

Taken together, these findings suggest that administration of E.melanin effectively alleviated PD-like symptoms induced by MPTP, thereby emerging as a potential therapeutic candidate for mitigating DaNs loss in PD.

### Administration of E.melanin alleviated MPTP-induced astrocytic activation and engulfment of synapses

**Fig. 3:**
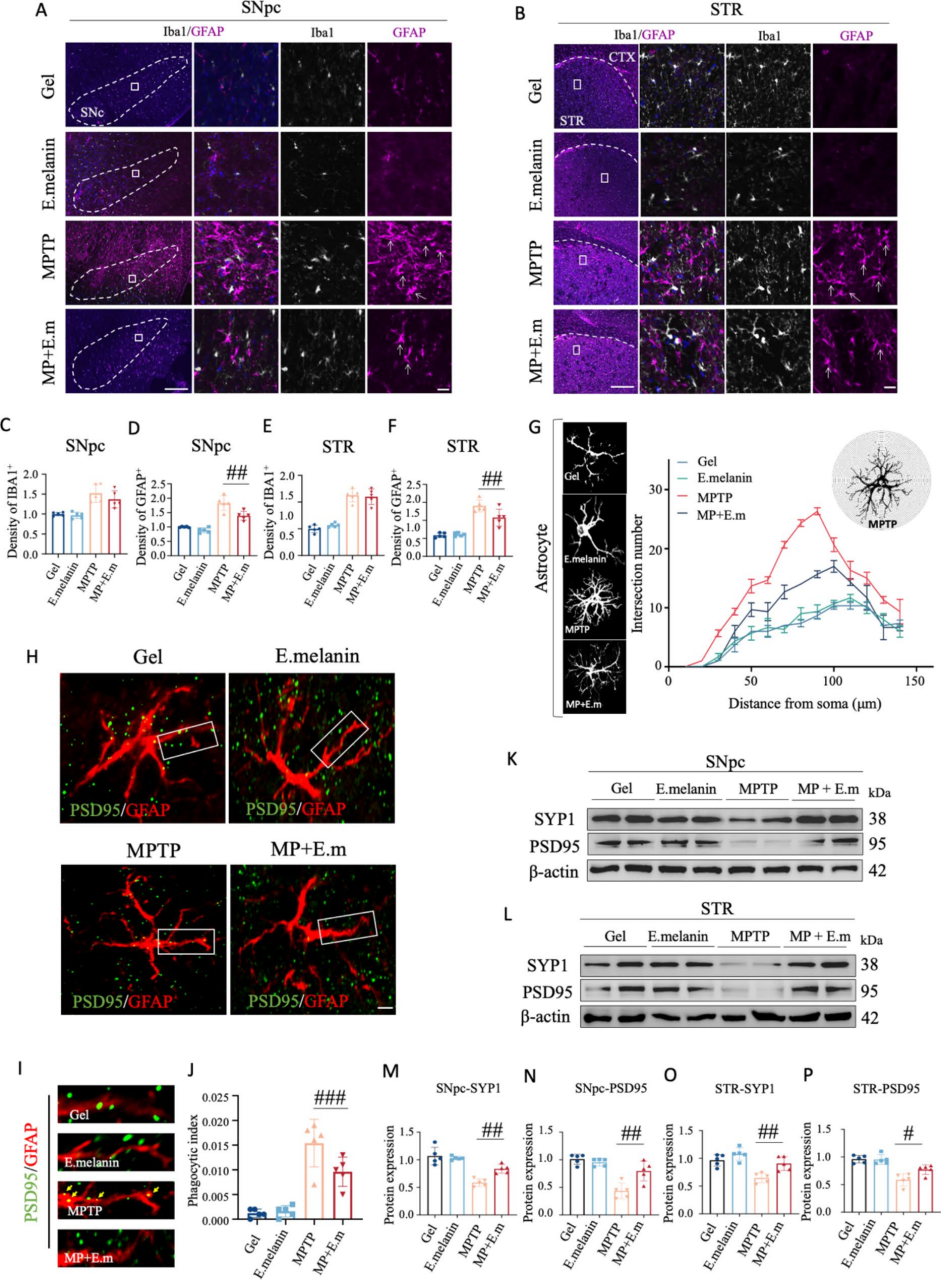
Administration of E.melanin mitigated MPTP-induced astrocytic activation and engulfment of synapses. (**A**) Typical immunofluorescent staining of Iba1 and GFAP in SNpc. White arrows indicate the activated GFAP. Scale bar: 250 μm. (**B**) Typica immunofluorescent staining of Iba1 and GFAP in STR. White arrows indicate the activated GFAP. Scale bar: 250 μm. (**C**) - (**F**) Quantifications of fluorescent intensity of Iba1 and GFAP in SNpc (panels (**C**) and (**D**)) and STR (panels (**E**) and (**F**)). (**G**) Sholl analysis of GFAP. Left panel: typical enlarged GFAP staining of astrocyte; Middle panel: distribution of intersection numbers from the Sholl analysis; Right panel: typical Sholl analysis of astrocyte. (**H**) Typical immunofluorescent staining of PSD95 and GFAP in STR. Scale bar: 10 μm. (**I**) Enlarged graphs of PSD95 and GFAP from panel (**H**). Scale bar: 5 μm. (**J**) Quantifications of phagocytic index of PSD95 in astrocyte. One-way ANOVA test, ***p < 0.001. (**K**) and (**L**) Western blot analysis of Synaptophysin 1 and PSD95 in SNpc (**K**) and STR (**L**). Abbreviations: SYP1: Synaptophysin 1; PSD95: postsynaptic density protein-95. (**M**) - (**P**) Quantifications of SYP1 and PSD95 in SNpc (panels (**M**) and (**N**)) and STR (panels (**O**) and (**P**)). One-way ANOVA test, *p < 0.05, **p < 0.01

To investigate the impact of E.melanin on glial cells, we conducted an analysis of glial activation in the SNpc and STR. Immunofluorescent staining revealed that both Iba1 and GFAP exhibited activation in MPTP-treated SNpc and STR (Fig. 3A and B). However, administration of E.melanin only reduced the number of GFAP-positive cells but not Iba1-positive cells (Fig. 3C-F). To assess structural plasticity, we quantified the intersection number of astrocytes using concentric circle (Sholl’s) analysis [23, 24]. The arborization of astrocytes was significantly increased in MPTP group compared to controls, while treatment with E.melanin abolished this effect (Fig. 3G), indicating a mitigated activation of astrocytes in PD mice. Furthermore, the expression levels of genes encoding neurotoxic reactive astrocyte markers (A1), namely *Ggta1*, *Cfb*, and *C3* were dramatically elevated in midbrains of MPTP-treated group (Supplementary Fig. 6A), whereas cytokine expression and other astrocyte subtype markers (A2) such as *Emp1*, *S100a10*, and *Cd109* were not significantly affected (Supplementary Fig. 6B).

Astrocytes are the most abundant cells in the brain and play essential roles in phagocytosis processes involving degenerating axons, apoptotic cells, and synapses [25, 26]. In accordance with this concept, we conducted an analysis of synaptic engulfment by astrocytes using PSD95 immunostaining and observed a decrease after treatment with E.melanin (Fig. 3H-J). Consistently, reductions in synaptic transmission markers synaptophysin-1 (SYP1) and postsynaptic density-95 (PSD95) also exhibited some degree of rescue in SNpc and STR following administration of E.melanin to MPTP-treated mice (Fig. 3K-P). Additionally, in primary cultured astrocytes, administration of M.E. successfully rescued MPP^+^-induced astroglia activation, engulfment of synapses and neurodegeneration (Supplementary Fig. 7). Furthermore, co-localization between SYP1 and PSD95 within STR was also restored upon E.melanin administration (Supplementary Fig. 8). These findings suggest that administration of E.melanin alleviated MPTP-induced astrocytic activation and reduced synaptic engulfment in PD mouse models.

### E.melanin treatment deactivated astrocytes and restored synaptic transmission pathways via single nuclear RNA sequencing of midbrain

**Fig. 4:**
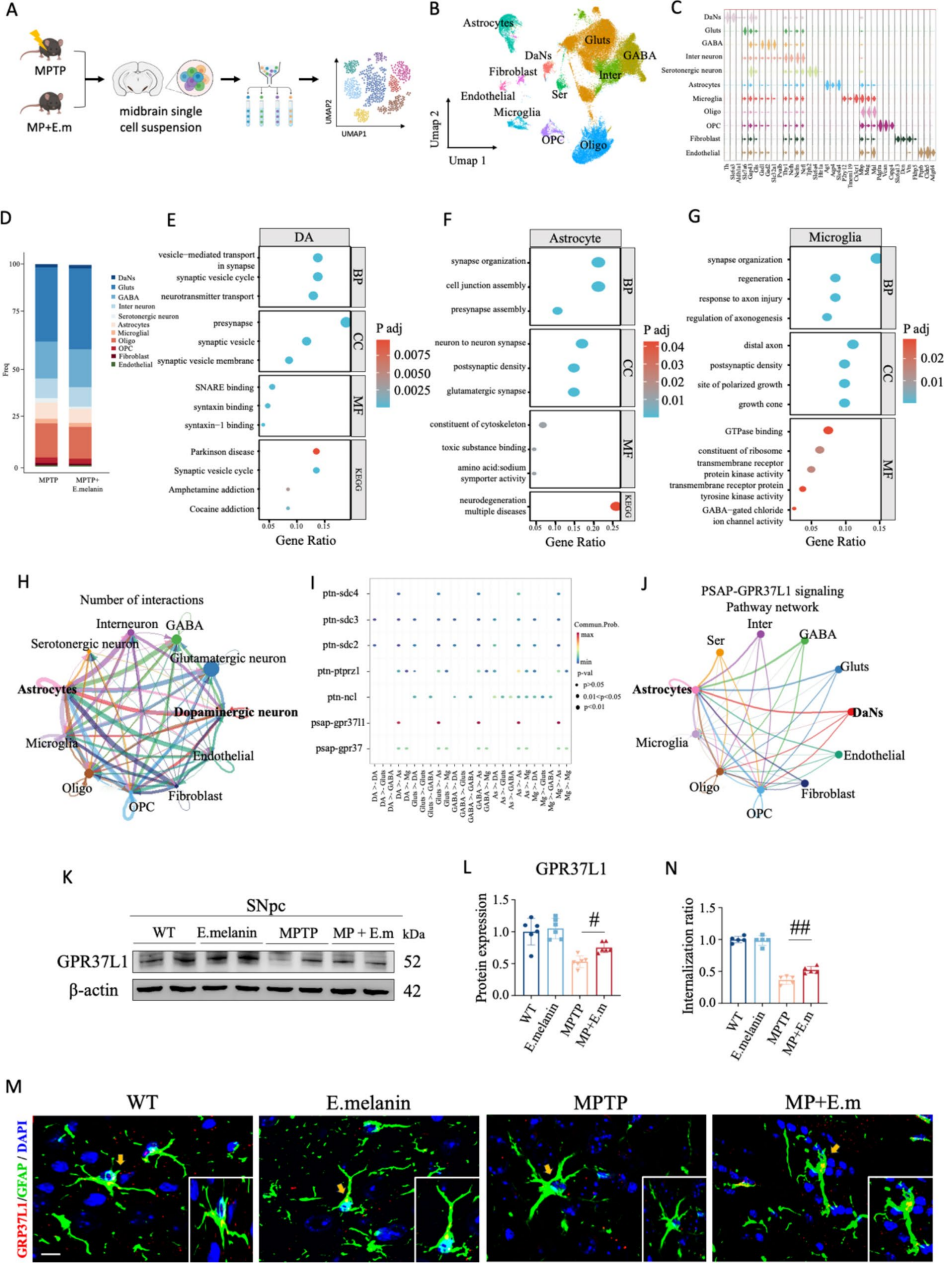
Single nuclear RNA sequencing of midbrain from MPTP and E.melanin-treated mice. (**A**) Cartoons illustrate the pipeline of PD mouse modeling and single nuclear RNA sequencing (snRNA seq) of midbrain. (**B**) The UMAP of 11 distinct cell types identified by unsupervised clustering. (**C**) Cell-type-specific expression of top DEGs identified in the snRNA-seq dataset. (**D**) Cell proportion changes in MPTP and E.melanin-treated midbrains. (**E**) Top GO and KEGG enrichment pathways identified in DaNs. (**F**) Top GO and KEGG enrichment pathways identified in astrocytes. (**G**) Top GO and KEGG enrichment pathways identified in microglia. (**H**) Number of cell-cell interactions identified via Cellchat. (**I**) Identification of signal pathways between neurons and glia cells. (**J**) Network of PSAP-GPR37L1 signaling pathway. (**K**) Western blot analysis of GPR37L1 in midbrain. (**L**) Quantifications of GPR37L1 from panel (**K**). One-way ANOVA test, *p < 0.05. (**M**) and (**N**) Immunofluorescent staining (**M**) and quantifications (**N**) of GPR37L1 and GFAP in midbrain. Yellow arrows indicate the localization of GPR37L1 in GFAP. One-way ANOVA test, **p < 0.01. Scale bar: 10 μm

To further investigate the underlying mechanisms responsible for the beneficial effects of E.melanin, we collected midbrains treated with MPTP and E.melanin for single nuclear RNA sequencing (snRNA-seq) analysis (Fig. 4A). Subsequently, we characterized the molecular taxonomy of these cells and identified 11 distinct transcriptional cell types in the SNpc (Fig. 4B and C, Supplementary Fig. 9). Notably, there was a significant change in astrocyte abundance following E.melanin treatment (Fig. 4D), indicating a transition to an inactivated state of astrocytes (Fig. 3). Gene Ontology (GO) and Kyoto Encyclopedia of Genes and Genomes (KEGG) enrichment analysis revealed that synaptic homeostasis-related pathways were enriched in DaNs, astrocytes, and microglia (Fig. 4E-G), suggesting a neuroprotective effect of E.melanin.

Furthermore, cell-cell communication analysis demonstrated that interactions between astrocytes and DaNs were more active than those involving microglia (Fig. 4H). Moreover, after administration of E.melanin, the PSAP-GPR37L1 pathway exhibited increased activation between astrocytes and neurons (Fig. 4I and J). Consistently, western blot analysis showed an elevated expression level of GPR37L1 in midbrains following E.melanin treatment (Fig. 4K and L), which was also confirmed by immunofluorescent staining of GPR37L1 and GFAP in SNpc (Fig. 4M and N). Additionally, the purified M.E. showed robust uptake efficiency and successfully rescued the expression levels of GPR37L1 in primary astrocytes (Supplementary Fig. 10). Collectively, these findings suggest that E.melanin treatment alleviates astrocytic activation and synaptic dysfunction possibly through the PSAP-GPR37L1 pathway; however, further investigation is required to elucidate the underlying molecular mechanisms.

**Fig. 5:**
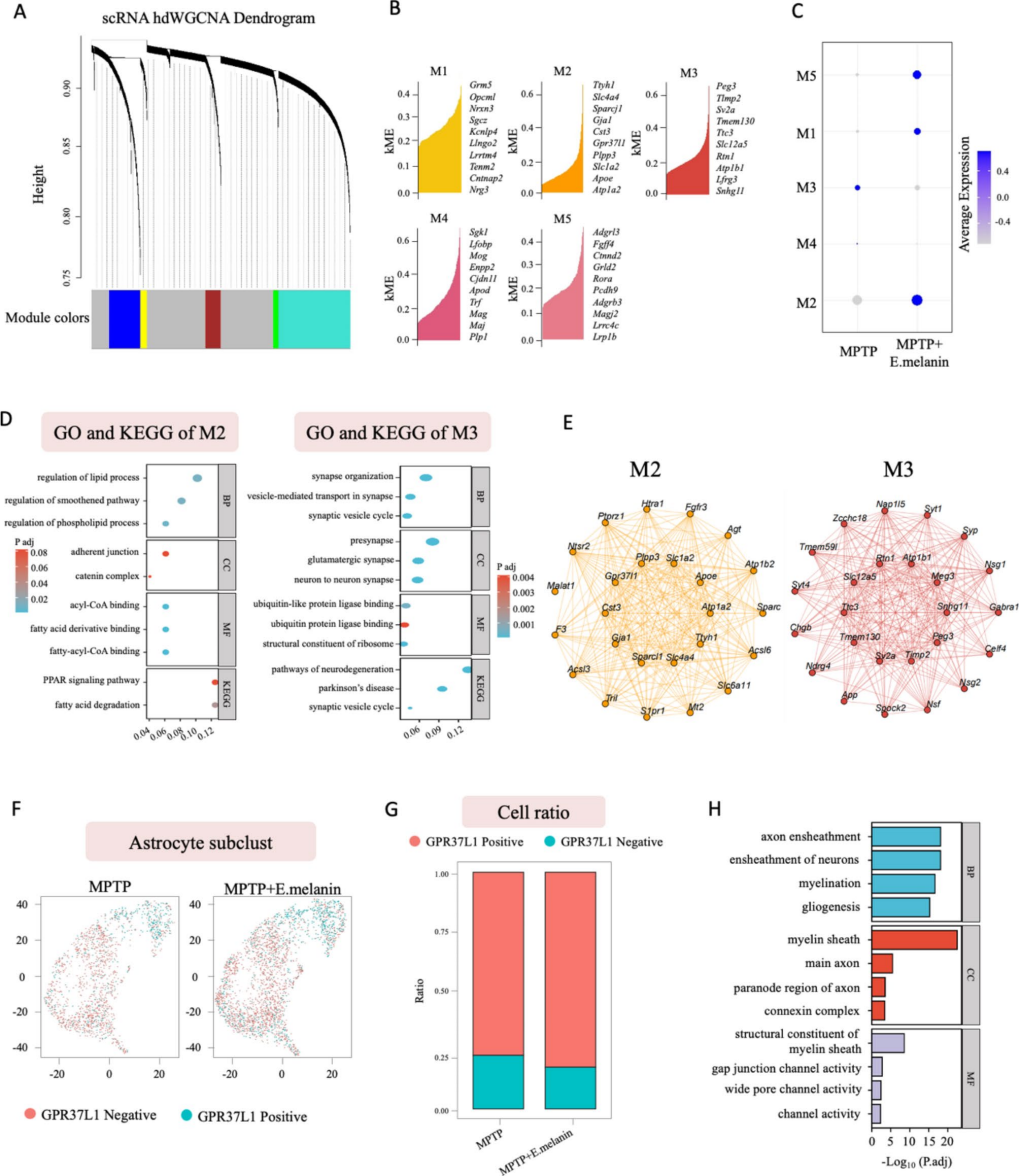
hdWGCNA analysis of astrocytes from snRNA seq dataset. (**A**) snRNA hdWGCNA dendrogram of astrocytes. (**B**) kME of hub genes identified in 5 modules. (**C**) Feature plot of hub genes of 5 modules identified in astrocytes. (**D**) GO and KEGG enrichment pathways of modules 2 and 3. (**E**) STRING analysis of hub genes of modules 2 and 3. (**F**) UMAP of astrocyte subcluster. (**G**) Ratio changes of GPR37L1 positive and negative cells in MPTP and E.melanin-treated midbrains. (**H**) Top GO and KEGG enrichment pathways identified in GPR37L1 positive astrocytes

Next, we performed WGCNA (weighted gene co-expression network analysis) analysis to find modules of highly correlated genes in astrocyte subcluster of the snRNA seq dataset (Fig. 5A). Summarizing such clusters using the module eigengene or an intramodular hub gene can identify candidate biomarkers or therapeutic targets [27]. In total, five functional modules were enriched in astrocytes (Fig. 5B), where modules 1, 2 and 5 were enriched in MPTP + E.melanin group, modules 3 and 4 were enriched in MPTP group (Fig. 5C). Additionally, GO and KEGG analysis identified that modules 3 was significantly enriched in the synaptic vesicle transmission and Parkinson disease pathways (Fig. 5D), suggesting it could serve a critical role in DaNs degeneration of MPTP-treated mice. Consistently, STRING analysis identified GPR37L1 as a major hub gene in module 2 (Fig. 5E). Additionally, in the astrocyte subcluster, we found an increase of GPR37L1-positive astrocyte in MPTP + E.melanin group (Fig. 5F), which was also confirmed in the cell ratio analysis (Fig. 5G). GO and KEGG analysis showed that GPR37L1-positive astrocyte was mainly enriched in myelination and myelin sheath pathways (Fig. 5H), indicating the repairment of myelin structure. In summary, these findings suggest that E.melanin administration relief the activation of astrocyte most likely through PSAP-GPR37L1 pathway, which needs to be validated via biochemical assays.

### Activation of GPR37L1 receptor rescued motor defects and DaNs loss in MPTP-induced PD mice

**Fig. 6:**
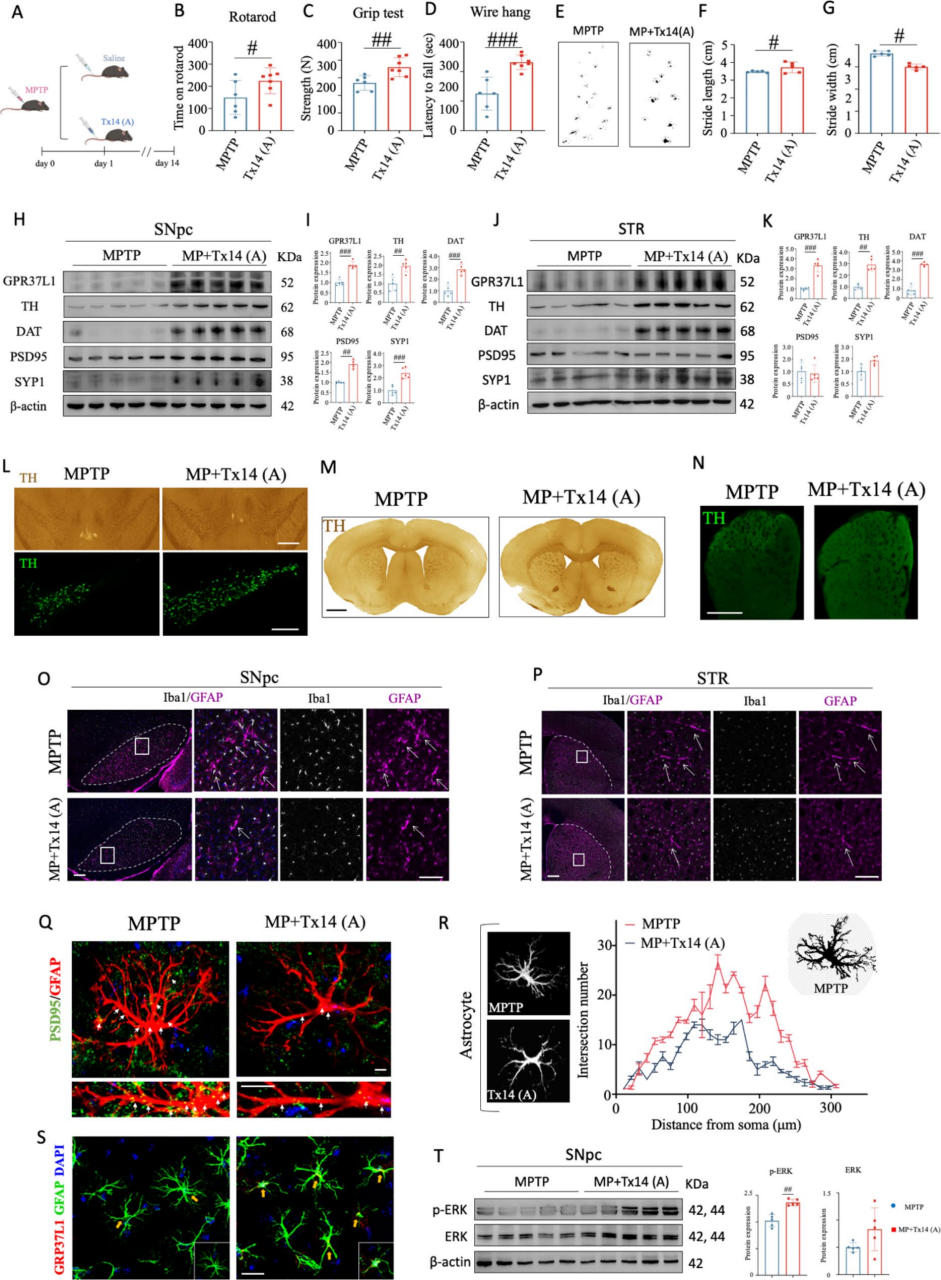
Activation of GPR37L1 receptor rescued motor defects and DaNs loss in MPTP-induced PD mice. (**A**) Schematic diagram illustrates the timepoints of MPTP and Tx14(A) injection. MPTP was intraperitoneally (i.p.) injected at 25 mg/kg for 7 days, where Tx14(A) was intravenously (i.v.) injected at 5 mg/kg for 14 days with 2-day intervals. (**B**) - (**D**) Quantifications of rotarod test (**B**), wire hang test (**C**), and grip strength test (**D**). Student’s *t*-test, *p < 0.05; **p < 0.01; ***p < 0.001. (**E**) Typical graphs of gait analysis in MPTP and Tx14(A)-injected mice. (**F**) and (**G**) Quantifications of stride length (**F**) and stride width (**G**). Student’s *t*-test, *p < 0.05. (**H**) Western blot analysis of GPR37L1, TH, DAT and synaptic markers in SNpc. (**I**) Quantifications of GPR37L1, TH, DAT, PSD95 and SYP1 in SNpc. Student’s *t*-test, **p < 0.01; ***p < 0.001. (**J**) Western blot analysis of GPR37L1, TH, DAT and synaptic markers in STR. (**K**) Quantifications of GPR37L1, TH, DAT, PSD95 and SYP1 in STR. Student’s *t*-test, **p < 0.01; ***p < 0.001. (**L**) Immunohistochemistry and immunofluorescent staining of TH in SNpc. Scale bar: 500 μm. (**M**) Immunohistochemistry staining of TH in STR. Scale bar: 500 μm. (**N**) Immunofluorescent staining of TH in STR. Scale bar: 200 μm. (**O**) Immunofluorescent staining of Iba1 and GFAP in SNpc. Scale bar: 200 μm. (**P**) Immunofluorescent staining of Iba1 and GFAP in STR. Scale bar: 200 μm. (**Q**) Immunofluorescent staining of PSD95 and GFAP. Scale bar: 10 μm. (**R**) Sholl analysis of GFAP. (**S**) Immunofluorescent staining of GPR37L1 and GFAP. Scale bar: 25 μm. (**T**) Western blot and quantifications of ERK and p-ERK in SNpc. Student’s *t*-test, **p < 0.01

Previous studies have suggested that activation of GPR37L1 receptor is facilitated by a peptide called Saposin C, along with its neuroactive fragments (prosaptide Tx14(A)), which have been shown to possess neuroprotective properties in various animal models by multiple research groups [28–30]. Subsequently, after a 7-day MPTP injection, Tx14(A) was administered for 14 days via intravenous injection to activate GPR37L1 (Fig. 6A). Based on behavioral analysis, Tx14(A) peptide effectively rescued motor coordination and balance deficits induced by MPTP, which was evidenced by results from rotarod test, wire hang test, grip strength test, and gait analysis (Fig. 6B-G).

Furthermore, administration of Tx14(A) consistently led to increased expression levels of GPR37L1, TH, DAT and synaptic markers in both SNpc and STR (Fig. 6H-K), suggesting that Tx14(A) may not only improve motor function but also promote dopaminergic neuron survival and function in PD mouse. Additionally, histological examination revealed reduced loss of DaNs in SNpc and STR brain regions involved in motor control after treatment with Tx14(A) (Fig. 6L-N), accompanied by reduced activation of astrocytes as well (Fig. 6O and P). This indicates that the neuroprotective properties of this peptide extend beyond just improving immediate symptoms but also provide long-term benefits for neuronal health. Consistently, Tx14(A) administration mitigated astrocytic activation and synaptic phagocytosis (Fig. 6Q-S). Moreover, activation of GPR37L1 by Tx14(A) resulted in increased expression levels of p-ERK in SNpc (Fig. 6T), suggesting that downstream signaling pathways associated with cell survival and neuroprotection being activated [28].

### Activation of GPR37L1 receptor alleviated αSyn pathology, and mitigated motor defects and DaNs loss in transgenic PD mice

**Fig. 7:**
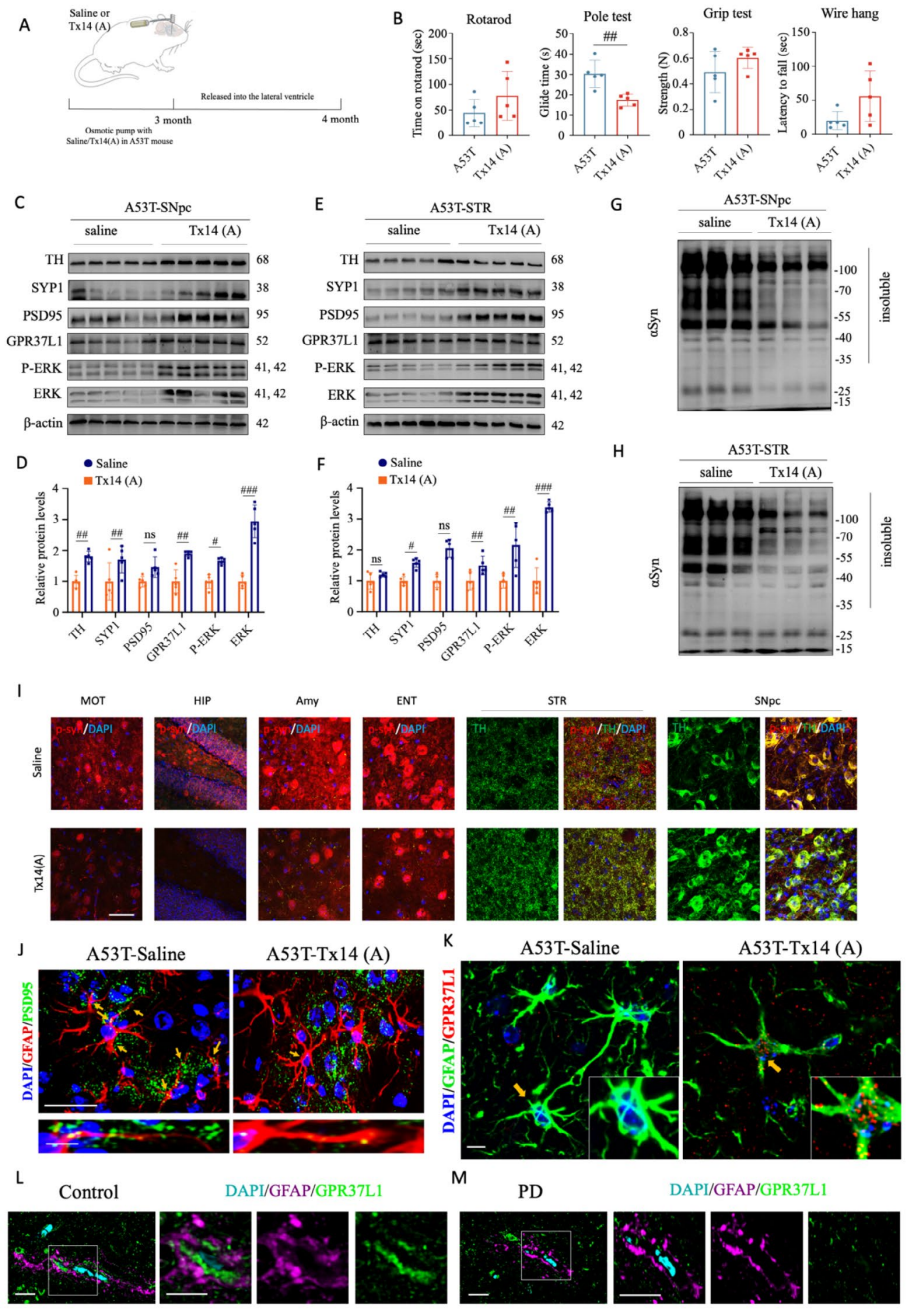
Activation of GPR37L1 receptor rescued αSyn pathology, motor defects and DaNs loss in αSyn A53T transgenic PD mice. (**A**) Schematic cartoon illustrates the infusion of saline or Tx14(A) peptide via intracerebroventricular (i.c.v.) route in αSyn A53T transgenic mice. (**B**) Animal behavioral analysis of rotarod, pole test, grip test, and wire hang post Tx14(A) administration for 1 month. Student’s *t*-test, ^**^p < 0.01. (**C**) - (**F**) Western blot analysis and quantifications of TH, SYP1, PSD95, GPR37L1, p-ERK, ERK in SNpc (**C** and **D**) and STR (**E** and **F**). One-way ANOVA test, *p < 0.05; **p < 0.01; ***p < 0.001. (**G**) and (**H**) Western blot analysis of p-αsyn in SNpc and STR of αSyn A53T transgenic mice. (**I**) Typical immunofluorescent staining of p-αsyn in MOT, HIP, Amy, ENT, STR and SN. The TH was stained in STR and SN (right two panels). MOT: motor cortex; HIP: hippocampus; Amy: amygdala; ENT: entorhinal cortex; STR: striatum; SN: substantia nigra. Scale bar: 100 μm. (**J**) Typical immunofluorescent staining of GFAP and PSD95 in SN. Scale bar: 50 μm. (**K**) Typical immunofluorescent staining of GFAP and GPR37L1 in SN. Arrows indicate the phagocytosis of synapses in astrocytes. Scale bar: 20 μm. (**L**) and (**M**) Typical immunofluorescent staining of GFAP and GPR37L1 in mesencephalon of healthy control (**L**) and patient diagnosed with PD (**M**). Please note, the intensity of GPR37L1 was dramatically decreased in GFAP-positive astrocyte of PD brain. Scale bar: 25 μm

Next, we investigated whether activation of the PSAP-GPR37L1 pathway can confer protection against αSyn pathology-induced motor impairments and loss of DaNs in transgenic PD mice. To test this hypothesis, we administered Tx14(A) peptide into 3-month-old αSyn A53T transgenic mice via intracerebroventricular (i.c.v.) osmotic mini-pump infusion for a duration of 1 month (Fig. 7A). Behavioral analysis revealed that Tx14(A) effectively rescued pole test performance, while rotarod test, grip strength test, and wire hang test showed some improvement but did not reach statistical significance yet (Fig. 7B).

Consistent with MPTP assays, administration of Tx14(A) peptide in transgenic PD mice effectively restored expression levels of TH, SYP1, PSD95, GPR37L1, ERK and p-ERK in SNpc and SYP1, PSD95, GPR37L1, ERK and p-ERK in SNPc and STR (Fig. 7C-F). Most importantly, the insoluble αSyn aggregates were reduced in the presence of Tx14(A) (Fig. 7G and H, Supplementary Fig. 11A and 11B). Furthermore, a decrease in p-αsyn staining was observed through immunofluorescent staining across several brain regions including motor cortex, hippocampus, amygdala, entorhinal cortex, STR, and SNpc (Fig. 7I, Supplementary Fig. 11C). Consistently, the intensity of TH staining was also increased in STR and SNpc (Fig. 7I, right two panels). Moreover, administration of Tx14(A) peptide decreased astrocytic phagocytosis of PSD95 while increasing astroglia localization of GPR37L1 (Fig. 7J and K, Supplementary Fig. 11D and 11E), Lastly, in brain specimens collected from healthy individuals as well as PD patients, it was found that GFAP-positive astrocytes exhibited reduced expression levels of GPR37L1 in PD brains compared to healthy controls (Fig. 7L and M, Supplementary Fig. 11F).

The collective findings suggest that activation of PSAP-GPR37L1 signaling pathway has potential for clinical management of PD by ameliorating astrocytic activation, reducing αSyn pathology, and rescuing DaNs loss in PD mouse models.

## Discussion

**Fig. 8:**
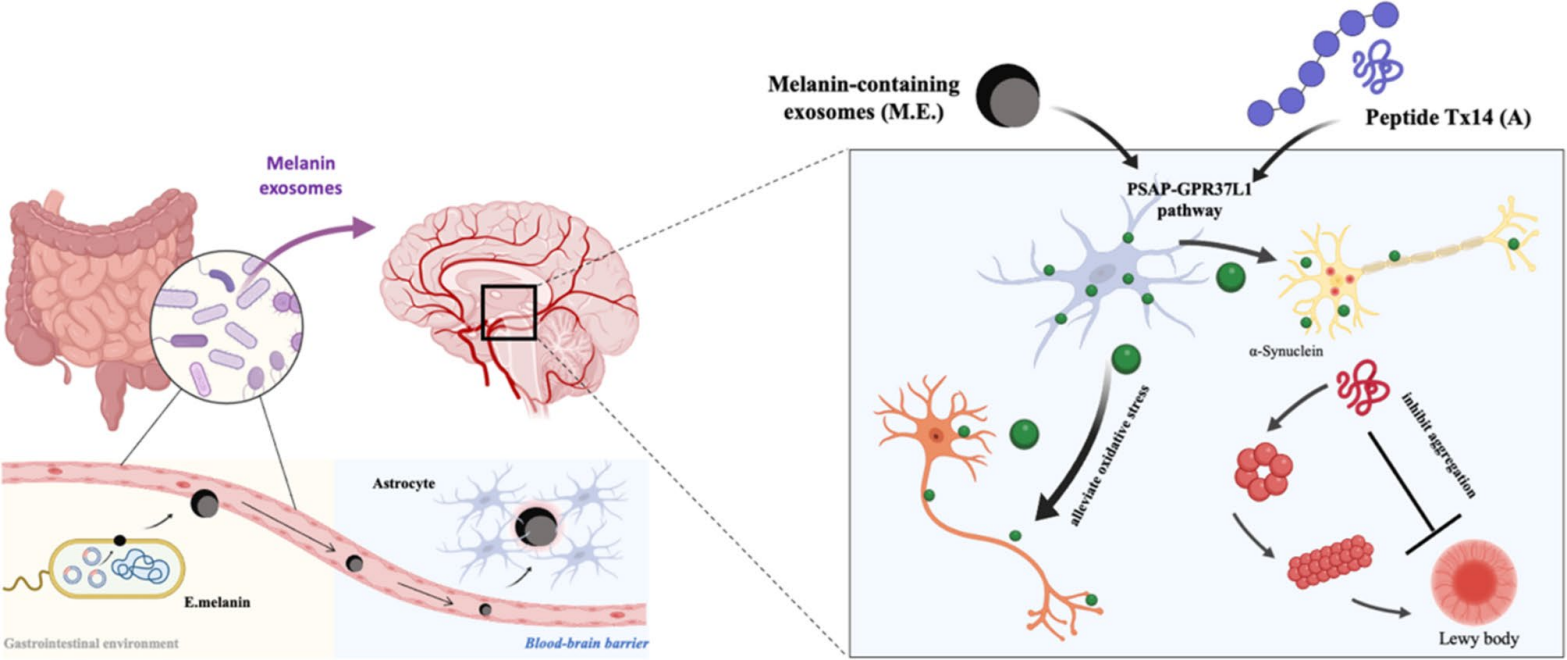
Cartoon summary of E.melanin activated the astrocytic PSAP-GPR37L1 pathway and alleviated the pathogenesis of PD. The genetically engineered E. coli was capable of secreting exosomes that contain melanin (E.melanin), which exhibited antioxidant properties in vitro, and mitigated activation of astrocyte via PSAP-GPR37L1 signaling pathway, thus reducing astroglia phagocytosis of synapses and alleviating dopaminergic neuron degeneration and αSyn aggregation in mice with PD

In the present study, the genetically engineered E. coli secrete exosomes containing melanin, which have been demonstrated to possess antioxidative properties both in cultured dopaminergic SH-SY5Y cells and in vivo. Mechanistically, snRNA seq data revealed that E.melanin specifically activates the PSAP-GPR37L1 signaling pathway within astrocytes, resulting in a reduction in synaptic engulfment. Consistently, administration of Tx14(A) peptide to activate GPR37L1 receptor rescued motor defects and attenuated DaNs loss in pharmacological and transgenic PD mouse models (Fig. 8).

Melanin is recognized as a potential scavenger of radicals; however, its antioxidative mechanisms remain poorly understood. In 2017, Liu et al. developed bioinspired melanin nanoparticles (MeNPs) that demonstrated efficacy against various reactive oxygen and nitrogen species (RONS), including O_2_-, H_2_O_2_, •OH, •NO, and ONOO- [19]. Furthermore, melanin-like nanoparticles also exhibited remarkable chelating capabilities towards diverse metal ions due to the presence of phenolic hydroxyl and amine groups on their surface [31]. Additionally, Zhao et al. discovered that melanin-like nanoparticles possess potent inhibitory effects on LPS-induced inflammation, making them promising nanodrugs for treating inflammatory diseases [32].

As for neural applications, overexpression of human tyrosinase in rat substantia nigra leads to age-dependent production of human-like neuromelanin within nigral dopaminergic neurons. However, intracellular accumulation of neuromelanin above a specific threshold is associated with an age-dependent PD phenotypes, including hypokinesia, formation of Lewy body-like structures, and neurodegeneration in the nigrostriatal pathway [17]. This suggests that intracellular levels of neuromelanin may determine the threshold for initiating PD. Additionally, due to its low solubility and limited availability, direct application of melanin as an anti-oxidative and anti-inflammatory strategy for treating PD and degeneration of dopamine-producing neurons remains unavailable. In this study, our engineered E. coli strain not only exhibits antioxidant properties but also shows potential in mitigating degeneration of DaNs and progression of PD. Most importantly, no significant side effects were observed. However, since we administered E.melanin to PD mice for only one-month, further investigation is needed to assess its long-term effect and bacterial toxicity.

The discovery of neuroprotective pathways is a top priority in the field of neurodegenerative diseases, particularly PD. Astrocytes, known for their natural neuroprotective properties, have the potential to enhance brain resilience. Among the G-protein coupled receptors expressed by astrocytes, two closely related receptors, GPR37L1 and GPR37, are of particular interest. Previous studies have shown that the PSAP-GPR37L1-GPR37 pathway plays a role in protecting neurons from oxidative stress and maintaining lipid homeostasis in dopamine neurons [28, 29]. Moreover, overexpression of PSAP via AAV protected against 6-OHDA and αSyn toxicity in wild-type rodents [33]. Furthermore, it has been shown that PSAP and Tx14(A) activate astrocyte motility through the activation of GPR37L1/GPR37 signaling pathway while inhibiting cAMP production specifically in astrocytes but not HEK293 cells [29]. In this study using snRNA seq analysis on midbrain samples administrated with MPTP or E.melanin, we demonstrate that PSAP-GPR37L1 signaling pathway is highly enriched in astrocytes. Additionally, activation of GPR37L1 by Tx14(A) leads to increased expression of p-ERK in SNpc suggesting activation of cell survival and neuroprotection signaling pathways [28].

The activation of astrocytes, which leads to the degeneration of DaNs and subsequent release of inflammatory cytokines, is associated with the pathogenesis of aging and various diseases including cancer, coronary heart disease, AD, PD, and other neurodegenerative disorders. Therefore, this study has the potential to facilitate the development of safe and effective antioxidants for broad biomedical applications.

## Materials and methods

### Construction of genetically engineered E. Coli MG1655 strain

To replicate the melanogenesis process in prokaryotic host E. coli, we have engineered an ampicillin-resistant plasmid with constitutive promoters, TYR encoding fragments, and relevant cofactor fragments assembled via Gibson assembly. Plasmid information can be referenced in our recent published literature [18]. Subsequently, the plasmid was introduced into the E. coli MG1655 bacterial strain, facilitating the development of the bacterial strain. Bacterial cells bearing the transformed plasmids were identified using LB agar plates supplemented with ampicillin (50 µg/mL).

### Melanin production

When L-tyrosine substrate was introduced into culture medium, the initially colorless solution rapidly transformed into an orange hue as dopachrome was generated. Subsequent addition of Cu^2+^ led to the gradual fading of the orange color, resulting in a brown color, indicating the progression of dopachrome polymerization into melanin.

### Extraction and purification of melanin exosomes

The genetically modified E.coli strain was supplemented into 10 mL of LB with the addition of ampicillin (50 µg/mL), CuS0_4_ (40 µg/mL), and L-tyrosine (0.5 mg/mL) for overnight incubation. The supernatant with melanin exosomes were then collected by centrifugation (7,000 r.p.m., 10 min).

### Particle size measurement

Dilute the melanin exosomes collected from overnight cultivation with pure water by 10 times. Then add it to the instrument (NanoSight NS300, Malvern Panalytical) according to the user manual.

### Total antioxidant capacity analysis with FRAP method

The principle of FRAP method for measuring total antioxidant capacity is that under acidic conditions, antioxidants can reduce Ferric pyridinyltriazine (Fe^3+^-TPTZ) to produce blue Fe^2+^-TPTZ. Then, measuring the blue Fe^2+^-TPTZ at 593 nm can obtain the total antioxidant capacity of the sample (Beyotime, S0116). Dilute the melanin exosomes collected from overnight cultivation with pure water by 0, 2, 4, 8 times. Then follow the instructions for operation.

### Total antioxidant capacity analysis with a rapid ABTS method

ABTS is oxidized to green ABTS^•+^ under the action of appropriate oxidants. The production of ABTS^•+^ is inhibited in the presence of antioxidants. The total antioxidant capacity of the sample can be determined and calculated by measuring the absorbance of ABTS^•+^ at 414–734 nm (Beyotime, S0121). Dilute the melanin exosomes collected from overnight cultivation with pure water by 0, 2,4,8 times. Then follow the instructions for operation.

### Cell viability analysis with cell counting kit-8 (CCK8) assay

WST-8 is a compound like MTT that can be reduced by some dehydrogenases in mitochondria in the presence of electron coupling reagents to produce orange yellow formazan. As cell proliferation increases, the color intensifies more rapidly; Conversely, higher cytotoxicity results in a lighter color. For identical cells, there is a linear correlation between color intensity and cell quantity. SH-SY5Y cells were plated in a 96-well plate at a density of approximately 50,000 cells per well. Following 24 h of culture with MPP^+^ (MCE, HY-W008719), melanin exosomes were added, and the cultivation was continued for another 24 h. Measure the absorbance (450 nm) 1 h later after adding the CCK8 (Beyotime, C0037) solution.

### Co-culture of E.melanin with SH-SY5Y cells

Plate E.melanin and SH-SY5Y cells on either side of the transwell plate, enabling them to interact indirectly through the micropores present on the membrane. The SH-SY5Y cells are divided into two groups: one group treated with the MPP^+^ drug and the other untreated control group. The E.melanin are divided into two groups: one group added with the Cu^2+^/Tyr and the other group unadded control group. They are paired with each other. After treating the SH-SY5Y cells with MPP^+^ for 12 h, introduce E.melanin for an additional 12 h of treatment, followed by JC-1 and TMRE staining.

### Analysis of mitochondrial membrane potential with JC-1 kit

When the mitochondrial membrane potential is high, JC-1 accumulates in the mitochondria matrix, forming polymers (J-aggregates) that emit red fluorescence. Conversely, when the mitochondrial membrane potential is low, JC-1 cannot accumulate in the mitochondria matrix and remains as monomers (J-monomers), emitting green fluorescence. The transition of JC-1 from red fluorescence to green fluorescence can be used as a detection indicator for early cell apoptosis. Stain the co-cultured SH-SY5Y cells following the provided instructions (Beyotime, C2006).

### Analysis of mitochondrial membrane potential with TMRE kit

Under normal conditions, the mitochondria contain a significant negative charge. TMRE, being a cationic probe, accumulates in the mitochondrial matrix and exhibits intense orange fluorescence upon cellular entry. During apoptosis, the loss of mitochondrial membrane potential results in the continuous opening of the mitochondrial permeability transition pore. This leads to the release of TMRE into the cytoplasm and a notable decrease in the intensity of red-orange fluorescence within the mitochondria. Evaluate alterations in mitochondrial membrane potential and the presence of apoptosis or necrosis based on the intensity of fluorescence signals. Stain the co-cultured SH-SY5Y cells following the provided instructions (Beyotime, C2001S).

### Animals

Adult male C57BL/6 mice were purchased from Shanghai Model Organisms Center, Inc., 8-week-old, and 25–28 g at the time of experiment and housed two to three per cage with ad libitum access to food and water during a 12 h light/dark cycle. All the experimental and surgical procedures were approved by Tongji University School of Medicine. A53T αSyn transgenic mice were purchased from Shanghai Model Organisms (B6/JGpt-Tg(hSNCA-A53T)62/Gpt), Shanghai, China. Unlike the commonly used A53T αSyn transgenic line M83 strain from the Jackson Laboratory, our current used A53T transgenic mice was driven by Thy promoter and exhibited robust behavioral defects and αSyn pathologies as soon as 3 months.

### Behavioral tests

To assess the potential positive impact of E. melanin or Tx14(A) treatment on behavioral impairments in an MPTP-induced PD mouse model, and the effects of Tx14(A) treatment on behavioral deficits in an α-syn overexpression-induced PD model, mice underwent evaluations using tests such as the rotarod test, wire hang test, grip test, pole test, and gait test. All tests were conducted following the procedures outlined in previous studies [34–36].

### Total protein extraction and Western blot analysis

Brain tissues were homogenized with a Polytron in ice cold T-PER Tissue Protein Extraction Reagent (thermo scientific; 78510) supplemented with protease and phosphatase inhibitors (Sigma; #P8340 and #P2850), sonicated and cleared by centrifugation (12,000 × r.p.m., 20 min, at 4 °C). Protein concentration in the supernatant was determined by BCA assay (Vazyme; E112-01). Protein (40 µg) in sample buffer (Beyotime; P0015N) was denatured by boiling at 95 °C for 5 min and separated on 12% sodium dodecyl sulfate poly acrylamide (SDS-PAGE) gels and transferred onto PVDF (Merck; 24937-79-9) by electrophoresis. Blots were blocked with 2% bovine serum albumin (Merck; 9048-46-8) for 1 h at room temperature and incubated with the primary antibody [(Anti-Tyrosine Hydroxylase; abcam; ab129991); (Anti-Dopamine Transporter; abcam; ab128848); (GPR37L1 Polyclonal Antibody; Invitrogen; PA5-106838); (Phospho-Erk1/2; CST; 4370); (Erk1/2; CST; 4695); (Synaptophysin1 antibody; SYSY; 101002); (PSD95 antibody; SYSY; 124002); (alpha-synuclein antibody; abcam; ab80627); (alpha-synuclein Phospho (Ser129) antibody; BioLegend; 825702); (Anti-beta Actin; abcam; ab8227)] overnight at 4 °C. After incubation overnight, blots were washed twice with TBST for 15 min each, then incubated with an HRP-conjugated secondary antibody (Goat Anti-Rabbit IgG H&L (HRP) (ab205718); Goat Anti-Mouse IgG H&L (HRP) (ab205719)) for 1 h at room temperature. Subsequently, blots were washed twice for 15 min each with TBST and stained using an Enhanced Chemiluminescence assay (thermo scientific; A38556).

### Immunohistochemistry and immunofluorescent staining

Mice were perfused with PBS and 4% PFA, and their brains were extracted. The brains were then fixed in 4% PFA overnight, transferred to a 20–30% sucrose gradient for cryoprotection, and subsequently embedded in OCT compound for freezing. Immunohistochemistry and immunofluorescence were conducted on 30-µm-thick consecutive brain sections. For histological examinations, brain sections were blocked with 5% goat serum in PBS containing 0.2% Triton X-100 and then incubated with TH, GFAP, Iba1, Synaptophysin1, PSD95, or GPR37L1 antibodies. Following three washes with PBS, brain tissues were incubated with the appropriate biotinylated secondary antibody, then treated with avidin-biotin complex (Zsbio, SP-9001), and visualized using 3,3’-diaminobenzidine (DAB) peroxidase substrate (Zsbio, ZLI-9018). For immunofluorescent experiments, the sections were rinsed in PBS and then exposed to a combination of Alexa Fluor 488, 594, and 647-conjugated secondary antibodies at room temperature for 120 min. Sections were analyzed using a confocal fluorescence microscope.

### Quantitative real-time PCR

Total RNA was extracted from tissues using TRIzol reagent. The cDNA was prepared by reverse transcription from mRNA (1 µg). mRNA expression levels were assessed using the SYBR Green PCR system. The relative expression of genes of interest was calculated by comparative Ct method and β-actin was used as an endogenous control. Sequences of the primers used for real-time qPCR are as follows: (*Il-1β*: F’ TGCCACCTTTTGACAGTGATG; R’ TGATGTGCTGCTGCGAGATT); (*Nf-κb*: F’ CTCTGGCACAGAAGTTGGGT; R’ TCCCGGAGTTCATCTCATAGT); (*Nlrp3*: F’ CCACATCTGATTGTGTTAATGGCT; R’ GGGCTTAGGTCCACACAGAA); (*Tnfα*: F’ CCCTCACACTCACAAACCAC; R’ ACAAGGTACAACCCATCGGC); (*C3*: F’ AGCTTCAGGGTCCCAGCTAC; R’ AGCTTCAGGGTCCCAGCTAC); (*Cfb*: F’ AGCTTCAGGGTCCCAGCTAC; R’ CCCCAAACACATACACATCC); (*Cgta1*: F’ CCCCAAACACATACACATCC; R’ TGACGTAAAATATGACCCGATGG); (*S100a10*: F’ CCAGGTTTCGACAGACTCTTC; R’ CCAGGTTTCGACAGACTCTTC); (*Cd109*: F’ CCAGGTTTCGACAGACTCTTC; R’ GAGTGTGAGCACCCGAAACTT); (*Emp1*: F’ GTTGGTGCTACTGGCTGGTC; R’ GTTGGTGCTACTGGCTGGTC); (β-actin: F’ CACGATGGAGGGGCCGGACTCATC; R’ TAAAGACCTCTATGCCAACACAGT).

### Hematoxylin-eosin staining and melanin staining

Hematoxylin-eosin (H&E) staining was performed in 5-µm-thick paraffin-embedded tissue sections. The Fontana-Masson Stain (Melanin Stain) (Abcam, ab150669) was used to stain melanin granules in 5 μm thick paraffin-embedded tissue sections. This staining technique is based on the capacity of melanin to chelate metals, consequently reducing silver nitrate to a visible metallic state. Briefly, paraffin tissue sections were deparaffinized and rehydrated by incubating at 60°C for 10 min, followed by multiple washes in xylene (3 times, 5 min each) and a series of ethanol washes (100%-95%-70%-H_2_O, 5 min each). Distilled water was used to rinse the samples between each step.

### Assessment of blood samples

Blood should be mixed with anticoagulants thoroughly during collection. The fully automatic biochemical analyzer measures a specific chemical component in serum or plasma based on the principle of photoelectric colorimetry. For example: blood routine testing and five classification blood cell analysis.

### Single nuclear RNA sequencing

In total, six mice (three MPTP induced PD model and three E.melanin treated PD model for single-nucleus RNA sequencing) were used. After the mice were sacrificed, the whole brain was separated carefully. The midbrain part of the brain was isolated. The isolation protocol mainly comes from the published 10x Genomics protocol for nucleus isolation. To reduce the impact of batch effects and variations in behavioral conditions on clustering, Seurat, which employs canonical correlation analysis and mutual nearest neighbor analysis, was utilized to combine all samples. Dimensionality reduction through Principal Component Analysis (PCA) was conducted on the integrated dataset. UMAP plots were generated for visualizing the clusters and sub-clusters. Identification of Differentially Expressed Genes (DEGs) involved the following criteria: (1) genes must exhibit at least a 1.5-fold overexpression in the target cluster, (2) genes must be expressed in over 25% of the cells in the target cluster, and (3) have adjusted *p*-value < 0.05. Gene Ontology (GO) and Kyoto Encyclopedia of Genes and Genomes (KEGG) enrichment analyses for the DEGs were then performed. Representative terms shown in the figures were selected within the top terms with the cumulative hypergeometric *P* < 0.05.

### Stereotaxic injection and implantation of osmotic pump

The stereotaxic coordinates used (Lateral ventricle) were: AP = + 0.3 mm; ML = -1.07 mm, DV = -2.50 mm relative to the bregma, according to the atlas of Paxinos and Franklin Mouse Brain Atlas. A capsule within an osmotic pump containing 100 µL of Prosaptide Tx14(A) (3 µg; bs-0088R, MCE, HY-P1342) was affixed under the skin on the back. A plastic cannula was inserted into the ventricle and connected to the osmotic pump for one month.

### Statistical analysis

All experiments were replicated at least three times. Off-line data analysis was performed using ImageJ (NIH), Prism 9 (Graph Pad software) and IGOR software (Wavemetrics). When appropriate, two-way ANOVA analyses of variance were used to test the interaction between two factors followed by Tukey’s post hoc. Comparison of groups was done by one-way ANOVA followed by Tukey’s multiple comparison test. Paired analysis was applied for repeated measurements and data originating from the same experimental subject. Outliers were removed by Grubb’s tests (alpha = 0.05). All data were presented as mean ± standard error of the mean (SEM). All tests were conducted using SPSS (Statistical Package for the Social Sciences) 13.0. Values of *p* < 0.05 were significant.

## Electronic supplementary material

Below is the link to the electronic supplementary material.

Supplementary material 1

## Data Availability

No datasets were generated or analysed during the current study.

## Declarations

### Study approval

Mice were raised and maintained in a barrier facility. All animal experiments were reviewed and approved by the Institutional Animal Care and Use Committee of the Tongji University, and conducted according to institutional guidelines.
