# Supplementary material for: Genetically modified E. Coli secreting melanin (E.melanin) activates the astrocytic PSAP-GPR37L1 pathway and mitigates the pathogenesis of Parkinson’s disease
Source: J Nanobiotechnology. 2024 Nov 10;22:690. doi: 10.1186/s12951-024-02955-x (PMC11552183; doi:10.1186/s12951-024-02955-x)
Supplement: Supplementary file 1 — Supplementary material 1 [file 12951_2024_2955_MOESM1_ESM.docx]

**Supplementary figure legends**


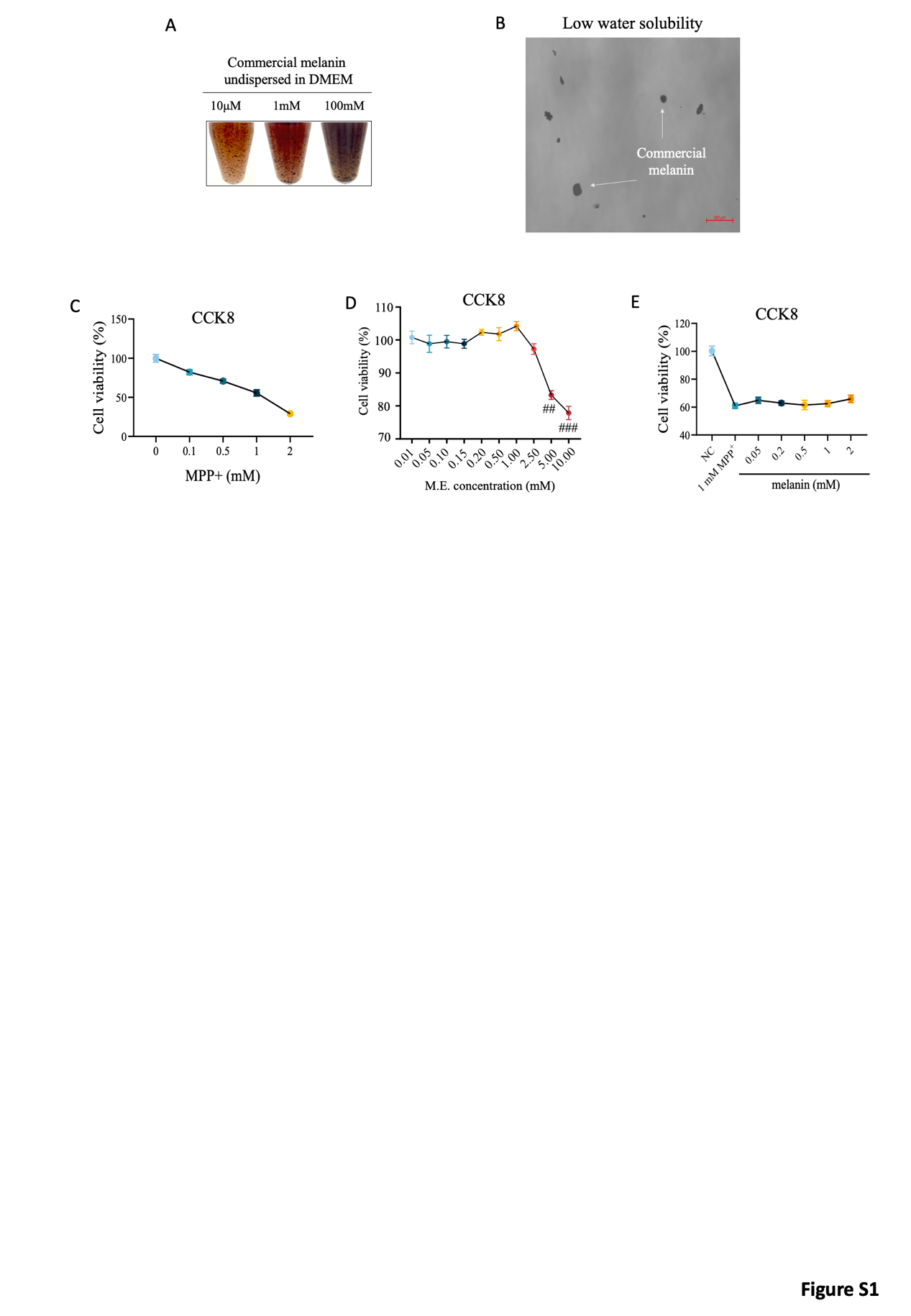
 **Supplementary Fig. 1: Characterization of a commercialized melanin.**

(A) Typical graphs of commercial melanin dissolved in DMEM at different concentrations.

(B) Image of undissolved melanin. Scale bar: 100 μm.

(C) CCK8 analysis of MPP^+^ on SH-SY5Y cells.

(D) CCK8 analysis of E.melanin on SH-SY5Y cells.

(E) CCK8 analysis of commercial melanin on SH-SY5Y cells.


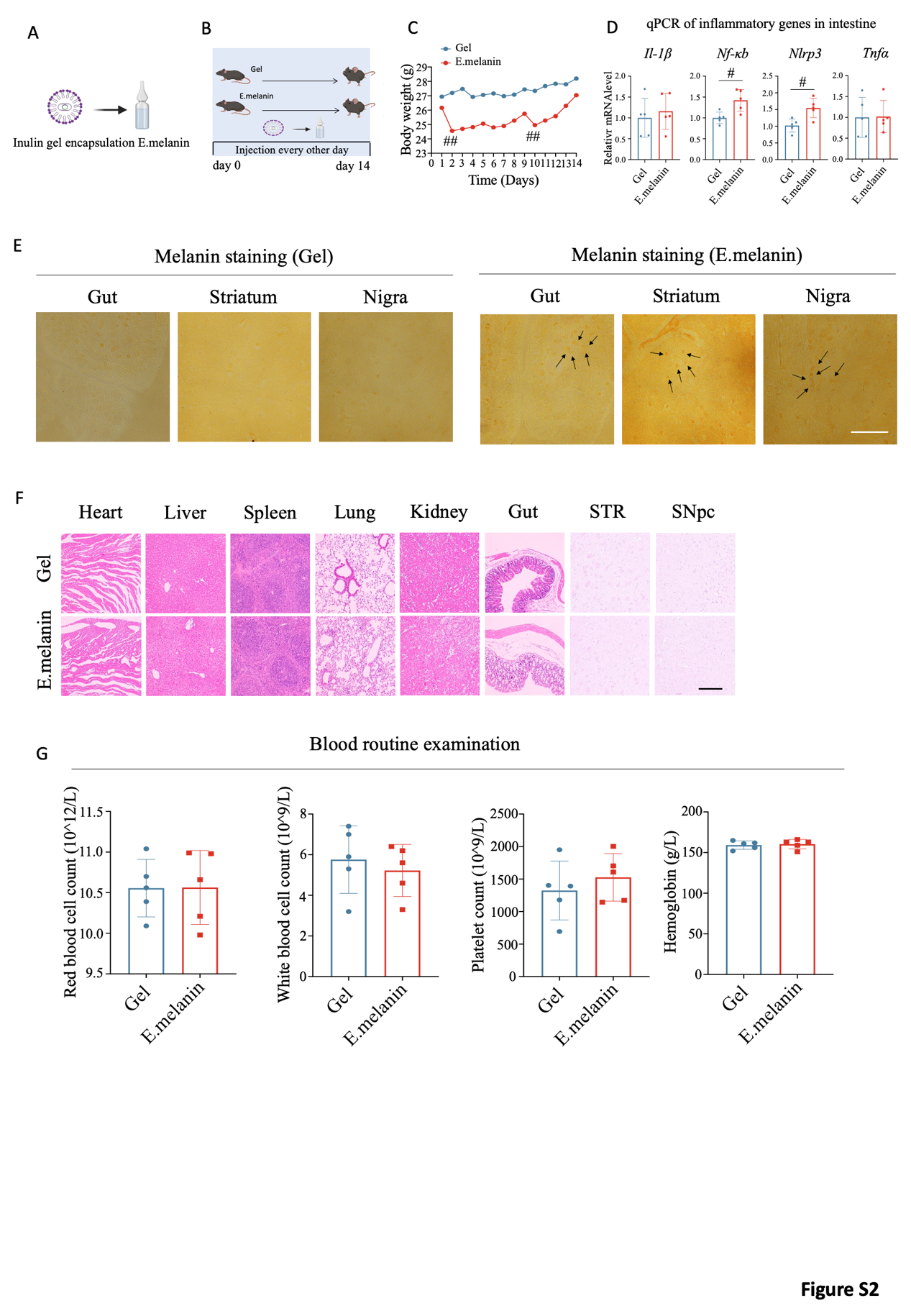
 **Supplementary Fig. 2: Characterization of E.melanin *in vivo*.**

(A) Schematic diagram of E.melanin encapsulation in inulin gel.

(B) Diagram of intragastric infusion of E.melanin in mice.

(C) Body weight of mice with or without E.melanin administration for 14 days with 2-day intervals.

(D) qPCR analysis of inflammatory genes in intestine. Student’s *t*-test, *p < 0.05.

(E) Immunohistochemistry staining of melanin in gut, STR, and SNpc with or without E.melanin administration for 2 weeks. Scale bar: 100 μm.

(F) Typical H&E staining of heart, liver, spleen, lung, kidney, gut, STR and SNpc with or without E.melanin administration for 2 weeks. Scale bar: 100 μm.

(G) Quantifications of blood routine examinations.


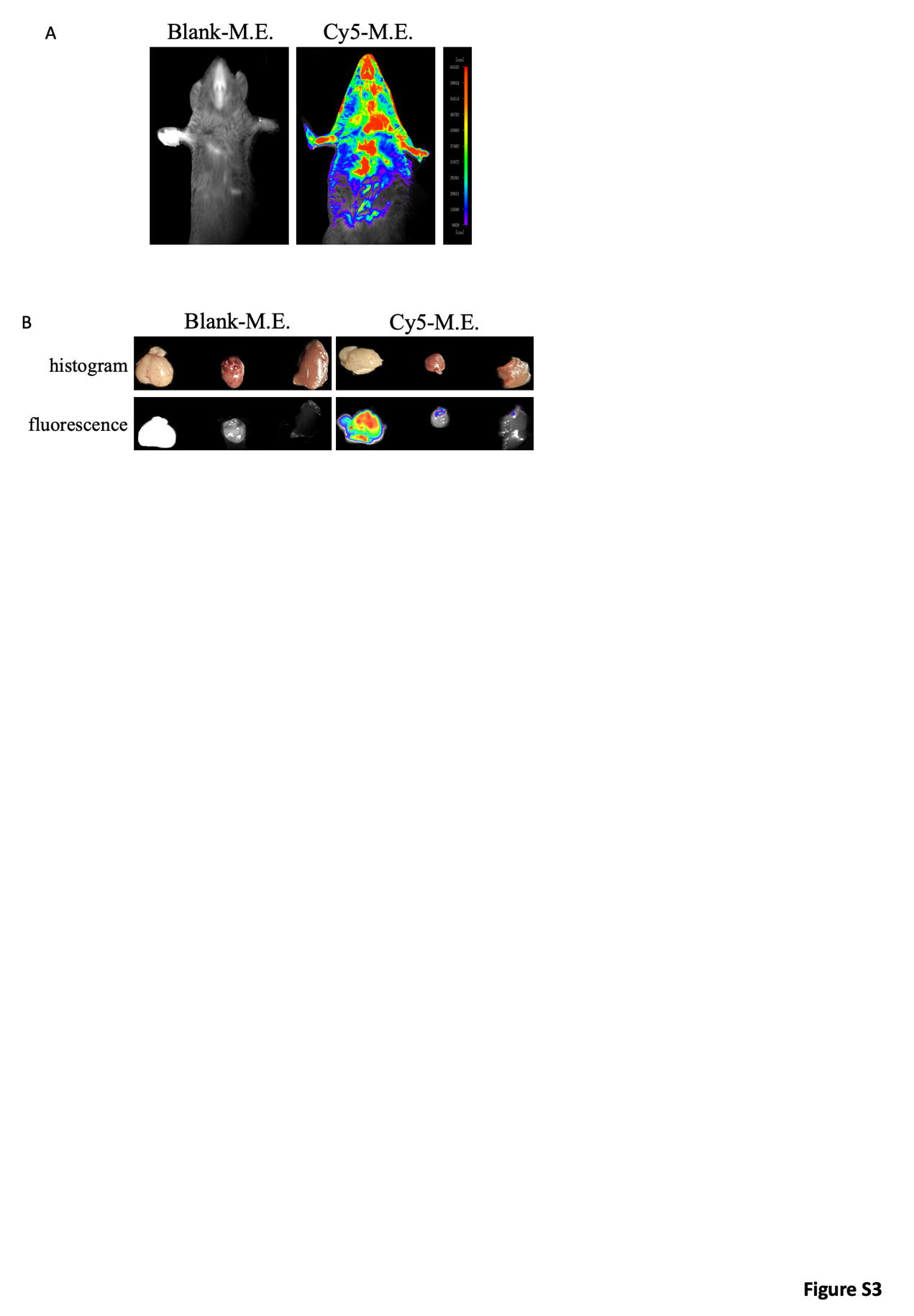


**Supplementary Fig. 3: Characterization of M.E. in different organs.**

(A) and (B) Tissue distribution in mice post Cy5-labelled melanin-containing exosomes (M.E.) administered via tail vein injection for 30 mins.

**
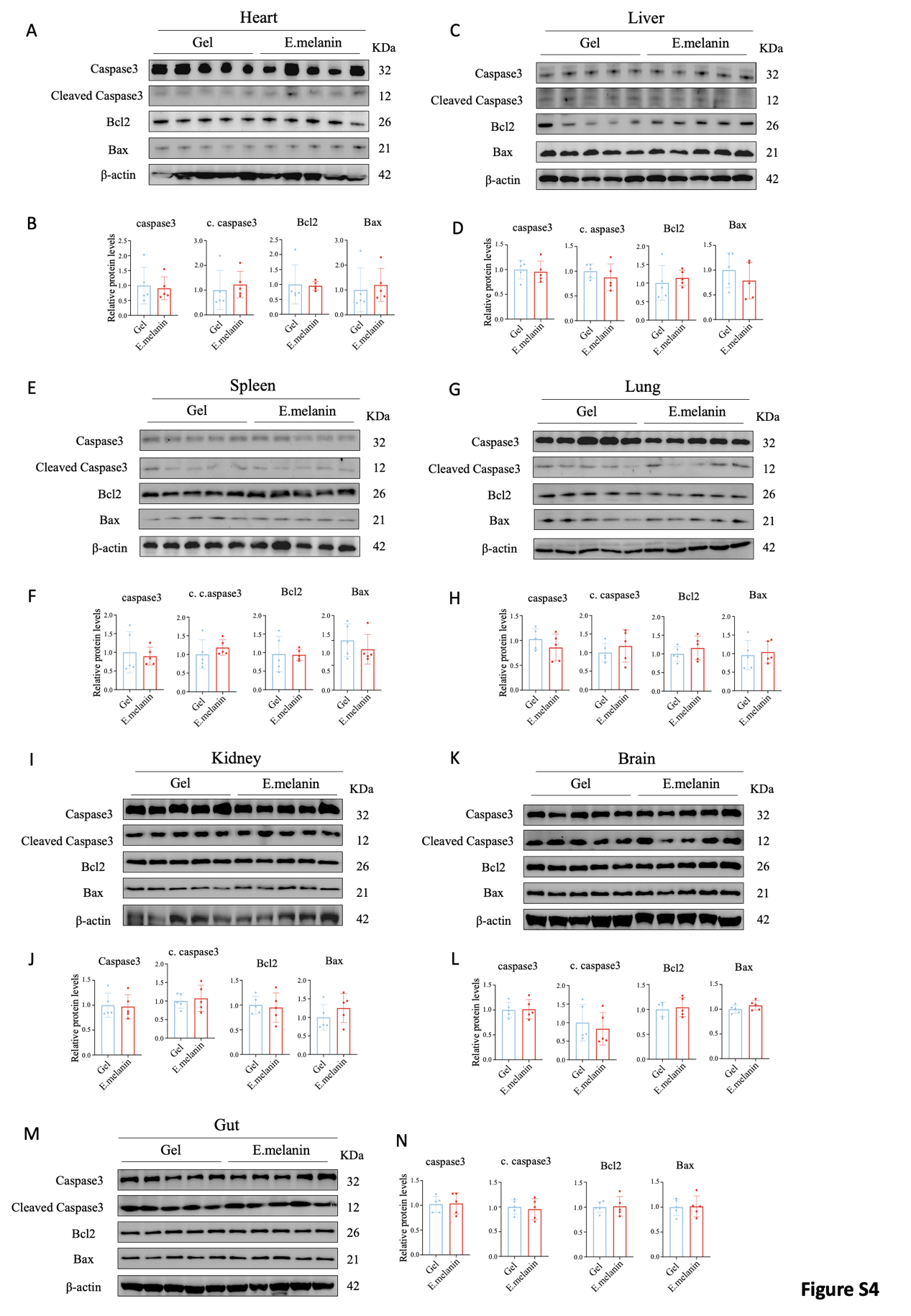
**

**Supplementary Fig. 4: Characterization of E.melanin administration-induced apoptosis *in vivo*.**

(A) Typical western blots of Caspase 3, cleaved Caspase 3, Bcl2 and Bax in heart with or without E.melanin treatment.

(B) Quantifications of Caspase 3, cleaved Caspase 3, Bcl2 and Bax in heart.

(C) Typical western blots of Caspase 3, cleaved Caspase 3, Bcl2 and Bax in liver with or without E.melanin treatment.

(D) Quantifications of Caspase 3, cleaved Caspase 3, Bcl2 and Bax in liver.

(E) Typical western blots of Caspase 3, cleaved Caspase 3, Bcl2 and Bax in spleen with or without E.melanin treatment.

(F) Quantifications of Caspase 3, cleaved Caspase 3, Bcl2 and Bax in spleen.

(G) Typical western blots of Caspase 3, cleaved Caspase 3, Bcl2 and Bax in lung with or without E.melanin treatment.

(H) Quantifications of Caspase 3, cleaved Caspase 3, Bcl2 and Bax in lung.

(I) Typical western blots of Caspase 3, cleaved Caspase 3, Bcl2 and Bax in kidney with or without E.melanin treatment.

(J) Quantifications of Caspase 3, cleaved Caspase 3, Bcl2 and Bax in kidney.

(K) Typical western blots of Caspase 3, cleaved Caspase 3, Bcl2 and Bax in brain with or without E.melanin treatment.

(L) Quantifications of Caspase 3, cleaved Caspase 3, Bcl2 and Bax in brain.

(M) Typical western blots of Caspase 3, cleaved Caspase 3, Bcl2 and Bax in gut with or without E.melanin treatment.

(N) Quantifications of Caspase 3, cleaved Caspase 3, Bcl2 and Bax in gut.


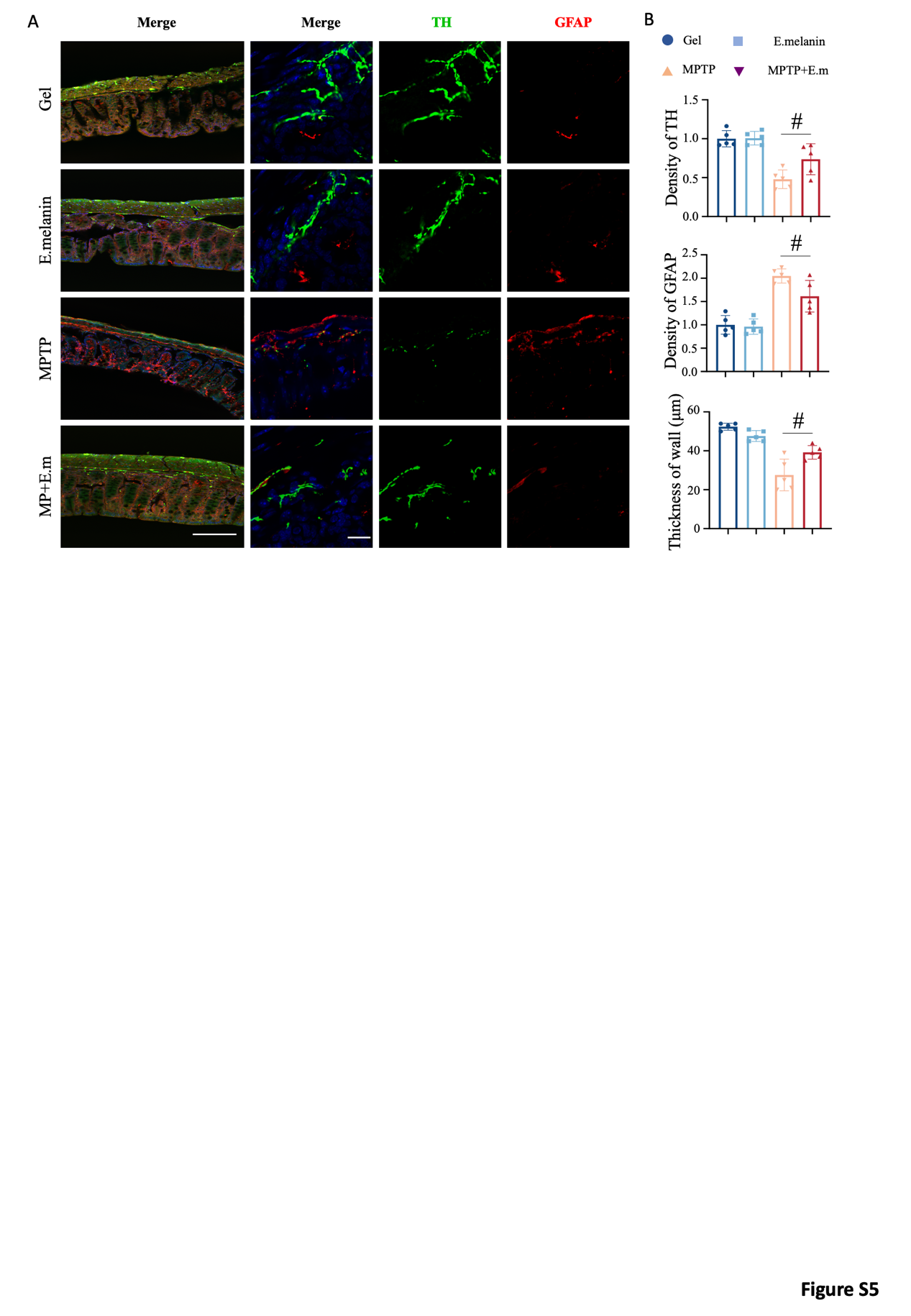


**Supplementary Fig. 5: Immunofluorescent staining of TH in intestines with different treatments.**

(A) Typical immunofluorescent staining of TH in intestine with different treatments. Scale bar: 100 μm and 25 μm.

(B) Quantifications of TH, GFAP intensity and thickness of gut wall. One-way ANOVA test, *p < 0.05.


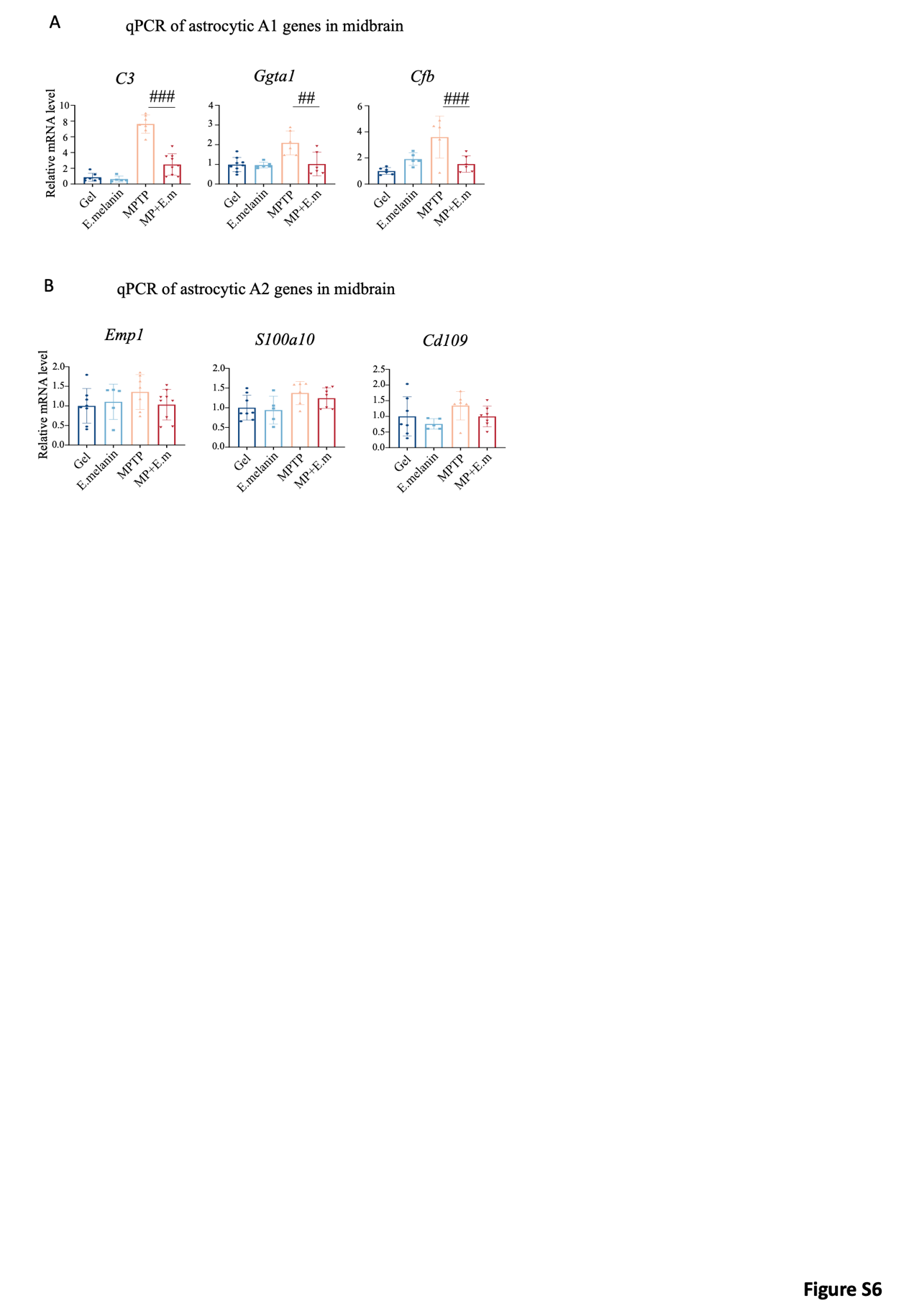


**Supplementary Fig. 6: qPCR analysis of A1 and A2 astrocytic genes in midbrain.**

(A) qPCR analysis of astrocytic A1 genes in midbrain. One-way ANOVA test, *p < 0.05; **p < 0.01; ***p < 0.001.

(B) qPCR analysis of astrocytic A2 genes in midbrain.


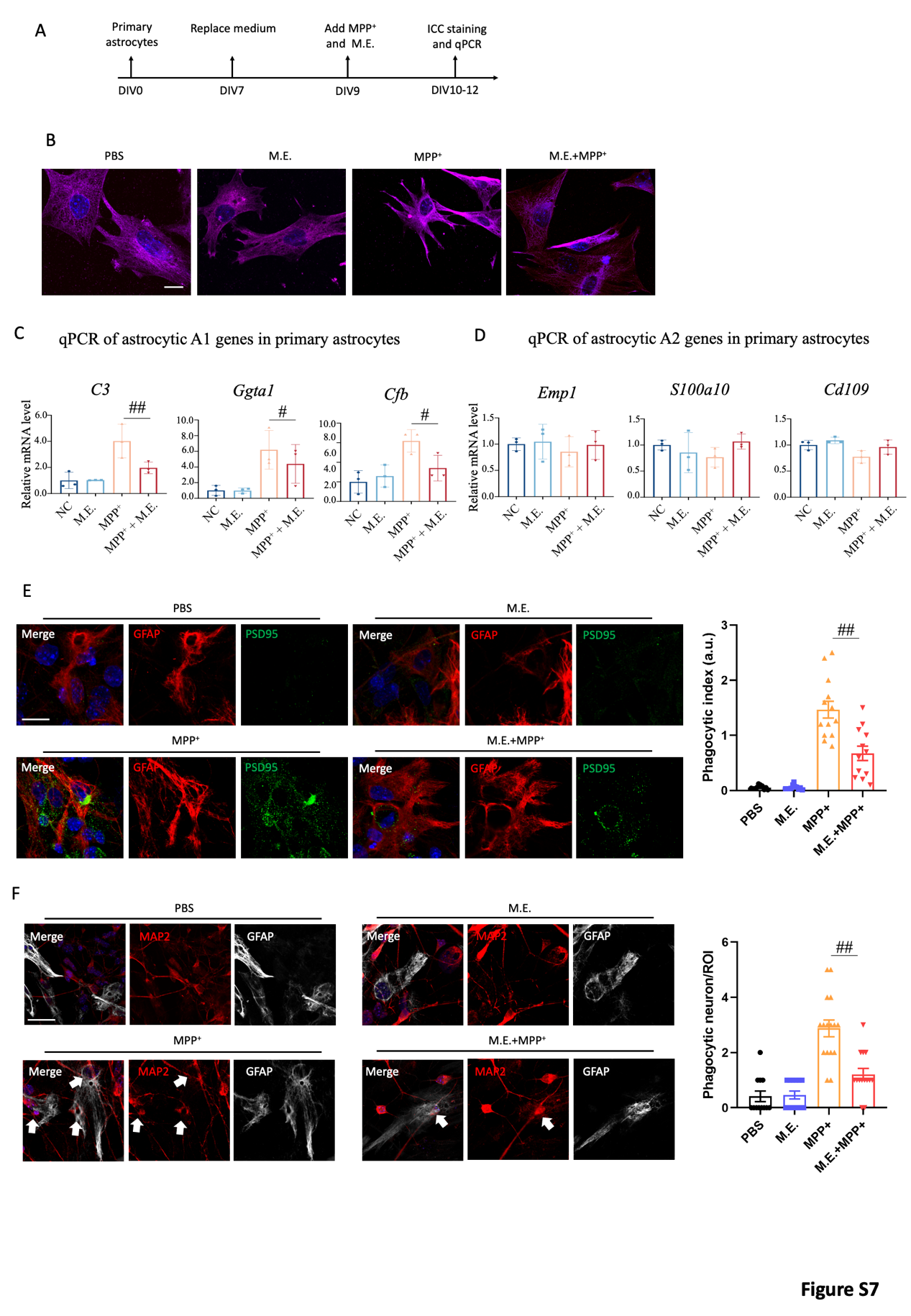


**Supplementary Fig. 7: Analysis of astrocytic activation and engulfment of synapses in primary cultures.**

(A) Schematic diagram of experimental protocols.

(B) Immunofluorescent staining of GFAP with distinct treatments. Scale bar: 10 μm.

(C) and (D) qPCR analysis of astrocytic A1 (C) and A2 (D) genes in midbrains. One-way ANOVA test, *p < 0.05; **p < 0.01.

(E) Immunofluorescent staining and quantifications of GFAP and PSD95 with distinct treatments. One-way ANOVA test, **p < 0.01. Scale bar: 50 μm.

(F) Immunofluorescent staining and quantifications of GFAP and MAP2 with distinct treatments. White arrows indicate the engulfed neurons by astrocytes. One-way ANOVA test, **p < 0.01. Scale bar: 50 μm.


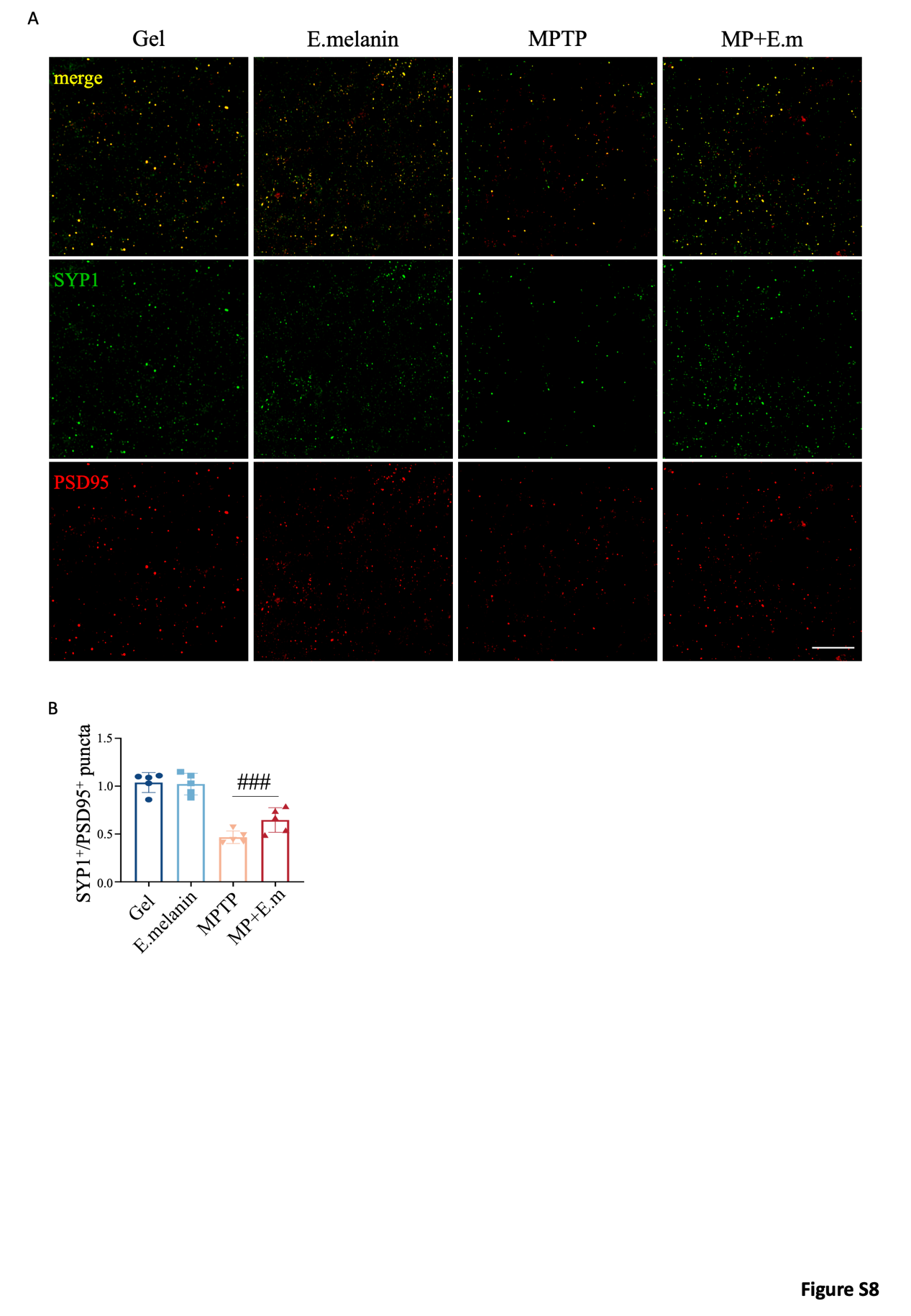
 **Supplementary Fig. 8. Immunofluorescent staining of SYP1 and PSD95 in striatum.**

(A) Typical immunofluorescent staining of synaptic markers, SYP1 and PSD95, in striatum. Abbreviations: SYP1, Synaptophysin 1; PSD95, postsynaptic density protein-95. Scale bar: 10 μm.

(B) Normalized number of SYP1^+^ and PSD95^+^ puncta. One-way ANOVA test, ***p < 0.001.


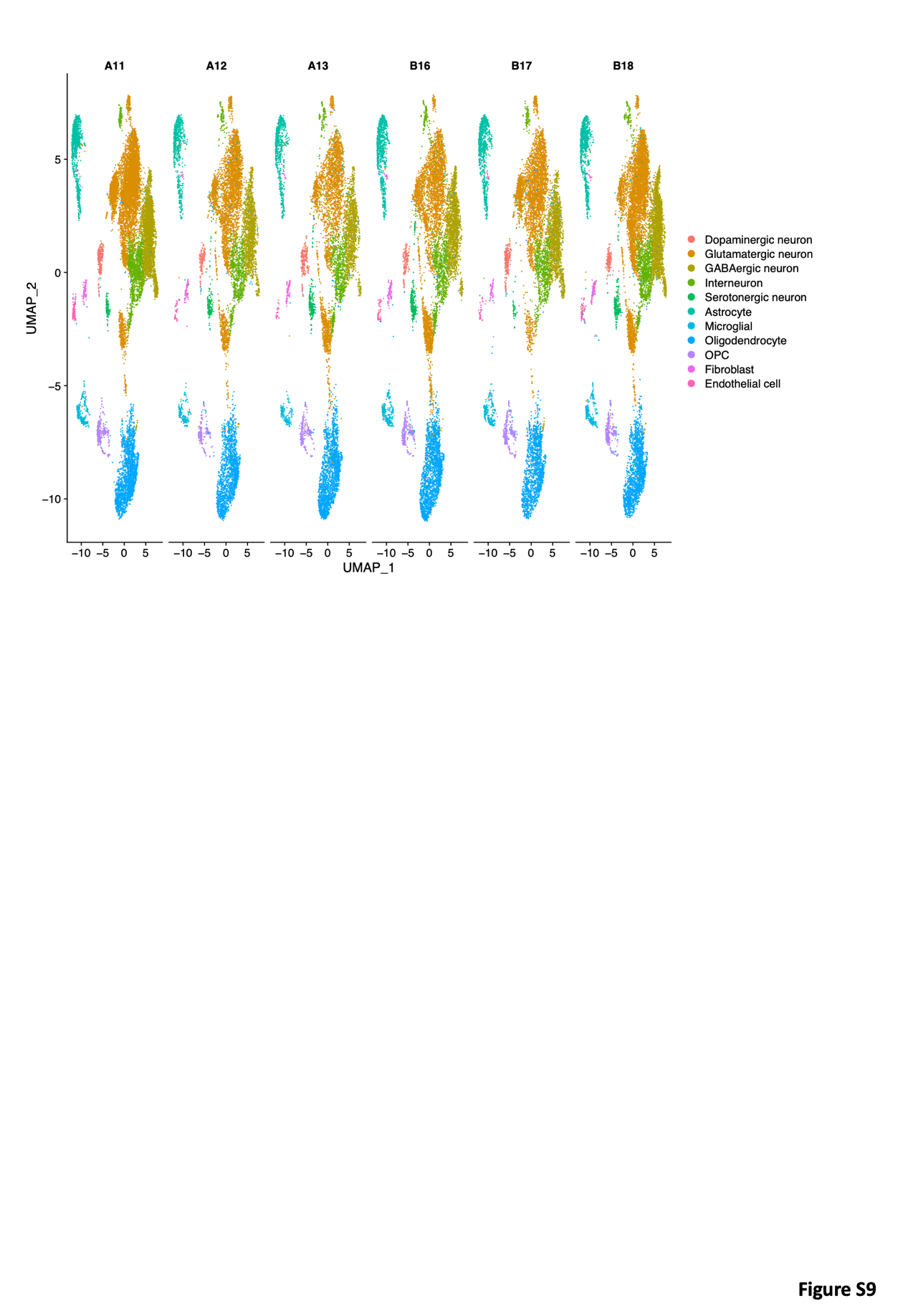


**Supplementary Fig. 9. UMAP distribution of different cell types in MPTP (A11, A12, A13)- and MPTP + E.melanin (B16, B17, B18)-treated midbrains.**


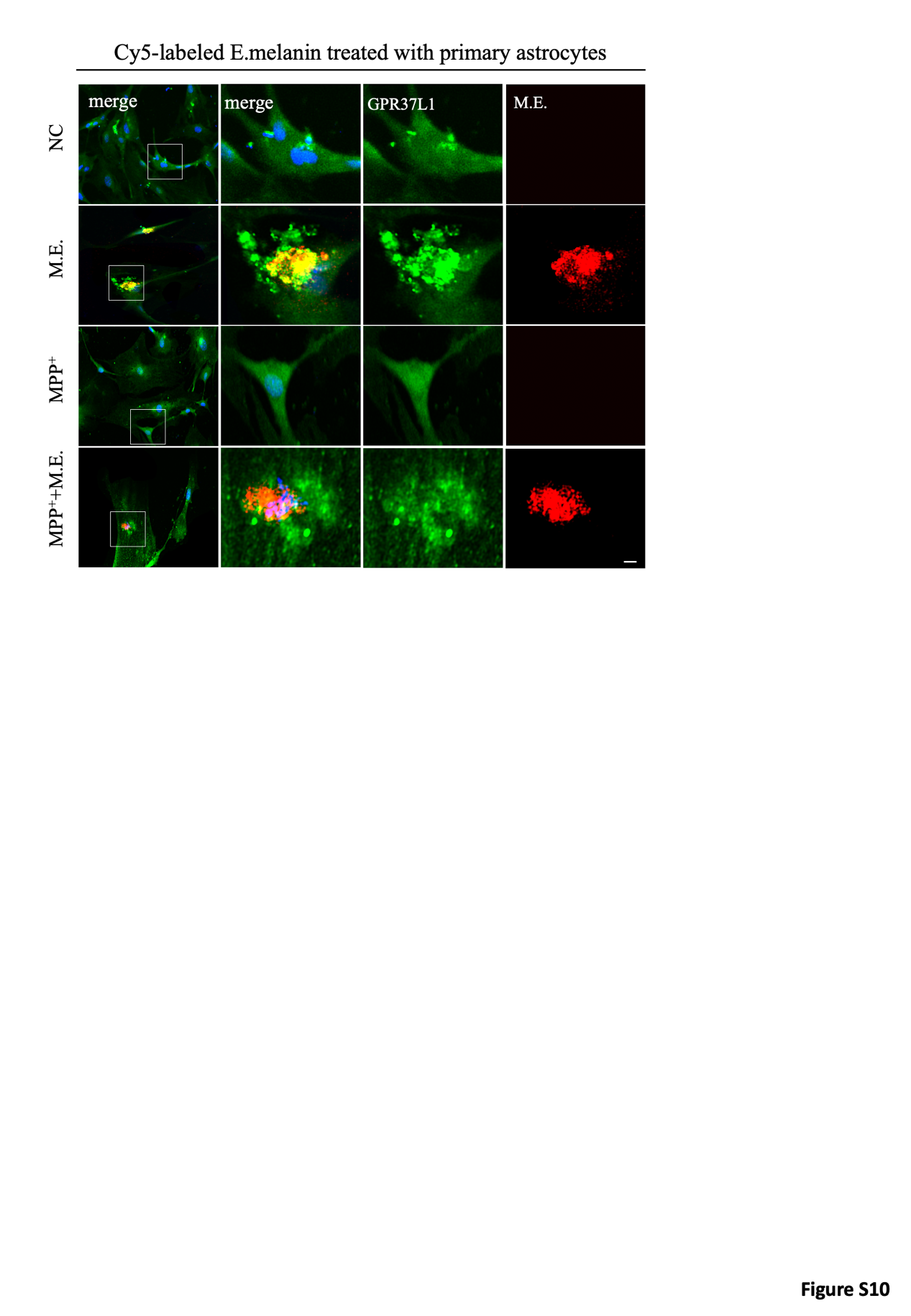


**Supplementary Fig. 10. Immunofluorescent staining of GPR37L1 and Cy5-labeled M.E. in primary astrocyte.**

Primary astrocytes were pre-treated with Cy-5 labeled M.E. overnight, followed with MPP^+^ treatment for another day. Then, astrocytes were fixed and stained with GPR37L1. Scale bar: 10 μm.


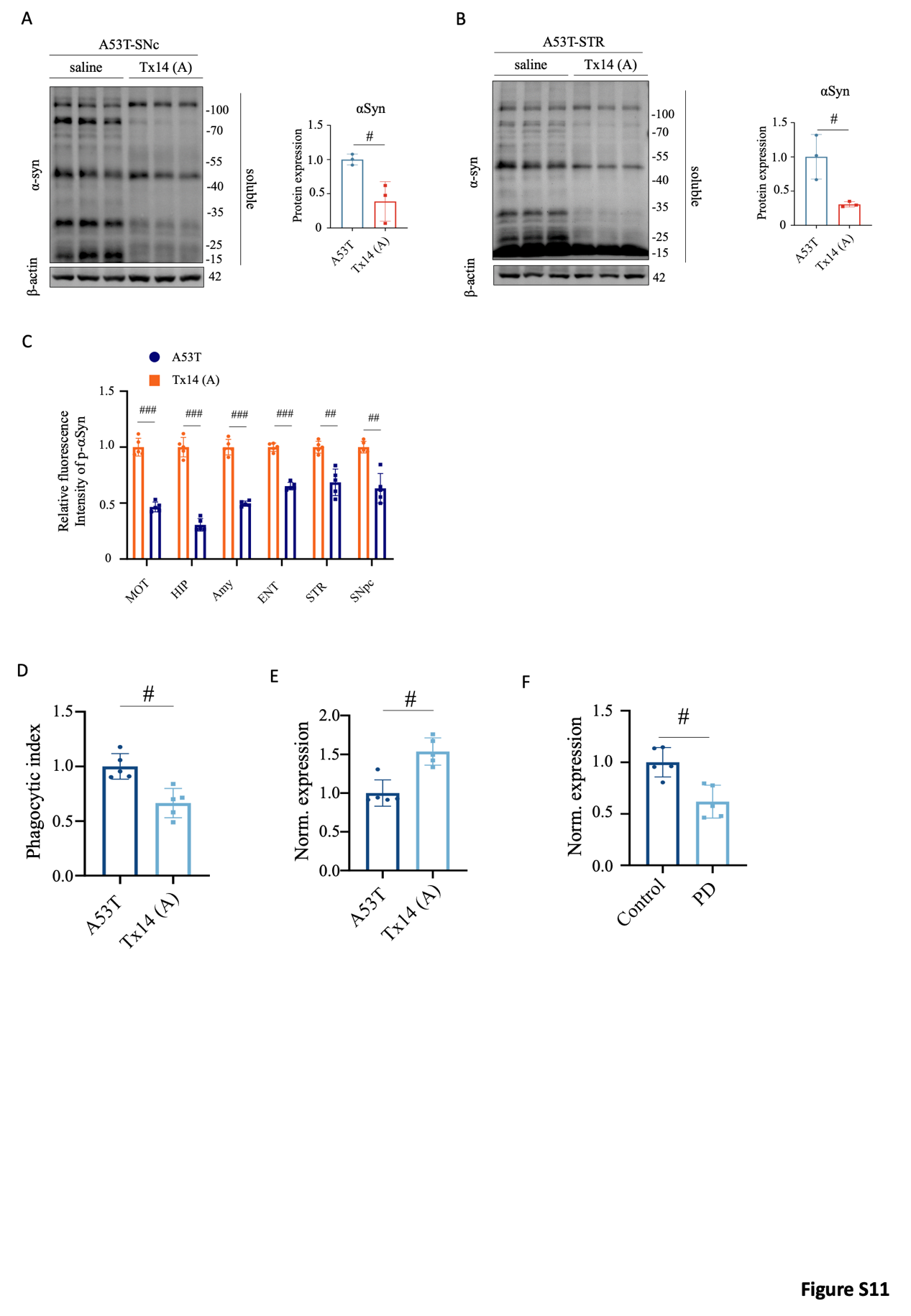


**Supplementary Fig. 11. Western blot analysis of soluble aSyn, and quantifications of aSyn in distinct brain regions, phagocytic index of PSD95, and normalized intensity of GPR37L1 in mice and PD brains.**

(A) and (B) Western blots and quantifications of soluble aSyn in SNc (A) and STR (B) after Tx14 (A) treatment in A53T aSyn transgenic mice. Student’s *t*-test, *p < 0.05; **p < 0.01.

(C) Quantifications of p-asyn in distinct brain regions. One-way ANOVA test, *p < 0.05; **p < 0.01; ***p < 0.001.

(D) Quantification of phagocytic index of PSD95. Student’s *t*-test, **p < 0.01.

(E) Quantification of normalized expression levels of GPR37L1 in A53T transgenic mice. Student’s *t*-test, *p < 0.05.

(F) Quantification of normalized expression levels of GPR37L1 in healthy controls and patients with PD. Student’s *t*-test, **p < 0.01.
